# Federated Quantum Machine Learning over Satellite Networks: Toward Scalable Distributed Quantum Classification

**DOI:** 10.3390/e28080924

**Published:** 2026-08-18

**Authors:** Juan Carlos Boschero, Rares Adrian Oancea, Luca Mazzarella, Hugo Doeleman, Simon Cramer

**Affiliations:** 1 Applied Cryptography & Quantum Algorithms, Netherlands Organisation for Applied Scientific Research, Anna van Buerenplein 1, 2595 DA Den Haag, The Netherlands; rares.oancea@tno.nl (R.A.O.); simon.cramer@tno.nl (S.C.); 2 Quantum Technologies, Netherlands Organisation for Applied Scientific Research, Stieltjesweg 1, 2628 CK Delft, The Netherlands; luca.mazzarella@tno.nl (L.M.); hugo.doeleman@tno.nl (H.D.)

**Keywords:** distributed quantum computing, quantum machine learning, federated learning, satellite quantum communication, quantum networks, distance-based quantum classification, neutral-atom quantum computing

## Abstract

Federated learning enables multiple organizations to collaboratively analyze data while retaining local control over their datasets, making it attractive for applications such as healthcare. Distributed quantum computing provides a natural framework for such workflows, allowing geographically separated quantum processors to execute joint computations using shared entanglement while preserving data privacy. In this work, we investigate the feasibility of satellite-enabled distributed quantum computing for federated quantum learning. As a representative application, we consider a distributed distance-based quantum classifier in which multiple parties contribute local data through quantum operations. To support this application, we develop a hybrid space-ground quantum network architecture in which satellites distribute entanglement between distant ground stations. The communication layer is combined with a noise-aware neutral-atom processor model, enabling a system-level analysis that captures both network and hardware constraints. Simulation results across varying network sizes, feature dimensions, and coherence regimes show that classifier performance is jointly determined by communication resources, processor noise, and data representation. In low-coherence regimes, decoherence destroys the classifier’s discriminative signal, whereas high-coherence regimes reveal limitations arising from feature-space conditioning and feature redundancy. These results demonstrate that satellite quantum networks could support distributed quantum learning over long distances, while highlighting the importance of coherence time, entanglement-distribution performance, and learning-aware data encoding for future large-scale deployments.

## 1. Introduction

Quantum networks are expected to extend quantum information processing from isolated devices to distributed systems in which remote nodes share entanglement, exchange quantum states, and jointly execute computational or communication tasks that are impossible, inefficient, or insecure using purely classical resources [1,2,3,4]. This vision is usually discussed under the broader concept of the quantum internet, where entanglement distribution enables applications ranging from quantum key distribution and clock synchronization to blind quantum computation, distributed sensing, and distributed quantum computing [2,3,5]. In parallel, quantum computing hardware remains limited by finite qubit counts, noisy gates, restricted connectivity, and decoherence, motivating distributed quantum computing (DQC) as a route to aggregate several smaller quantum processing units (QPUs) into a larger, logical computational resource [6,7]. In this setting, entanglement is not only a communication primitive, but a computational resource that allows non-local quantum gates to be implemented through local operations, measurements, and classical feed-forward [8,9].

A particularly relevant application domain for distributed quantum computing is privacy-preserving and federated quantum machine learning. In classical federated learning, multiple parties collaboratively train or evaluate a model while keeping raw data local, typically exchanging only model updates or aggregated statistics [10,11]. This paradigm is especially important in healthcare, where data are sensitive, regulated, and often distributed across institutions. However, classical federated learning still requires careful treatment of privacy leakage through gradients, model updates, or inference queries [11]. Quantum information processing offers a complementary route: because quantum measurements disturb quantum states and because distributed protocols can be constructed from shared entanglement and local operations, quantum networks may enable new forms of secure collaborative computation [5,12]. This motivates the study of distributed quantum machine learning (QML) protocols in which multiple data-owning parties contribute to a joint inference task without directly transmitting their raw feature vectors.

Within quantum machine learning, distance-based classifiers provide a useful primitive because they connect naturally to both kernel methods and nearest-neighbour-style reasoning. Schuld, Fingerhuth, and Petruccione introduced a quantum distance-based classifier in which classical feature vectors are amplitude-encoded into quantum states and compared through an interference circuit [13]. The central quantity estimated by the circuit is the overlap between two encoded states, which can be interpreted as a kernel value and converted into a Euclidean distance for normalized real-valued data [13]. Compared with more resource-intensive quantum machine learning proposals, this classifier is attractive because the inference primitive is conceptually simple: after state preparation, the distance information is extracted from interference and measurement statistics. Nevertheless, the practicality of the approach depends strongly on state-preparation overhead, shot noise, circuit depth, and the conditioning of the classical feature space.

Neumann and Wezeman extended this idea to a distributed quantum machine learning setting, showing how multiple parties can participate in a distributed distance-based classifier (DDBC) without explicitly revealing their local data [12]. In their construction, a server coordinates the computation while remote parties contribute controlled state-preparation operations associated with their local feature vectors. Non-local controlled operations are mediated using shared entanglement and classical communication, so that the distributed classifier inherits both the algorithmic structure of the centralized distance-based classifier and the resource constraints of distributed quantum computing [8,12]. This makes the protocol a natural candidate for federated quantum machine learning, as each party retains local control over its dataset and participates in a joint classification task without requiring direct exchange of the underlying training data.

The feasibility of such a protocol depends critically on the underlying quantum network. Direct long-distance optical-fiber transmission suffers from exponential attenuation, making intercity and intercontinental entanglement distribution challenging without quantum repeaters [14,15]. Satellite quantum communication provides a complementary approach because most of the optical path can occur through free space, where propagation losses scale more favorably than in fiber over long distances [16,17]. The Micius satellite experiment demonstrated entanglement distribution between ground stations separated by approximately 1200 km, showing that satellite links can outperform direct fiber transmission by many orders of magnitude in long-distance entanglement distribution scenarios [16]. More recent studies have further analyzed satellite quantum communication architectures, including hybrid space–ground networks, link-budget constraints, multiplexing, and repeater-like extensions based on memories and Bell-state measurements [17,18,19] (BSM). These developments make satellite networks a plausible infrastructure layer for future globally distributed quantum applications, including federated quantum machine learning.

However, satellite-enabled DQC is not determined by link availability alone. Distributed quantum algorithms require repeated non-local operations, and every such operation consumes entanglement, incurs classical communication latency, and may require quantum memories to buffer entangled states before use [14,15,19]. For a distributed classifier, these communication-layer constraints interact directly with algorithmic resource requirements: increasing the number of parties, training points, or feature dimension increases the number of controlled state-preparation operations, which in turn increases entanglement consumption and exposure to decoherence. Consequently, a realistic feasibility study must couple the learning algorithm, the satellite entanglement-distribution architecture, and the physical noise model of the quantum processors.

Neutral-atom processors based on Rydberg-mediated interactions are a compelling platform for this type of study because they combine scalable tweezer-array architectures, optical interfaces, and high-connectivity entangling operations [20,21]. Recent experiments have reported high-fidelity parallel entangling gates in neutral-atom arrays, with two-qubit gate fidelities around 99.5% on many atoms in parallel [22]. At the same time, Rydberg platforms exhibit characteristic physical noise processes, including spontaneous decay, off-resonant scattering, laser phase noise, dephasing, atom loss, and measurement assignment errors [20,21,22]. These mechanisms must be represented at the circuit level when evaluating long, distributed circuits, especially because communication latency and quantum memory storage times can dominate the physical duration of non-local gates.

In this work, we present a system-level feasibility study of satellite-enabled federated quantum machine learning. We focus on a distributed distance-based quantum classifier applied to a privacy-sensitive medical classification setting based on the Breast Cancer Wisconsin Diagnostic dataset [23,24]. The dataset serves as a representative clinical use case in which several hospitals or medical centers hold local patient-derived feature vectors and wish to collaboratively classify a query case without centralizing the underlying data. The goal is not to claim clinical deployment readiness, but to use a realistic privacy-sensitive dataset to evaluate how algorithmic performance depends on network size, feature dimension, shot budget, satellite link assumptions, and processor coherence.

Our architecture consists of geographically separated ground stations, each hosting a local quantum processor and quantum-memory interface, connected through a satellite-mediated entanglement distribution layer. Entangled photon pairs are generated and distributed through free-space optical downlinks, loaded into quantum memories, and consumed to implement the non-local controlled operations required by the distributed classifier. We model the communication layer using probabilistic entanglement generation, multiplexing, latency, and memory-dephasing effects. We then combine this with a circuit-level neutral-atom noise model that includes duration-dependent relaxation and dephasing, gate infidelity, two-qubit Rydberg error channels, and measurement assignment noise. This integrated modeling approach allows us to study not only whether the classifier works ideally, but also where it fails when realistic physical and network constraints are included.

The main contribution of this paper is to connect three research areas that are often studied separately: satellite quantum communication, distributed quantum computing, and quantum machine learning. Prior work on quantum distance-based classification established the interference-based classifier primitive [13]; prior work on distributed quantum machine learning showed how such primitives can be distributed across parties using entanglement [12]; and prior work on satellite quantum communication demonstrated and modeled long-distance entanglement distribution [16,17,19]. In contrast, the present study evaluates the full stack needed for an application-level task: a privacy-preserving distributed classifier executed over a satellite-enabled quantum network with realistic processor and memory noise. This systems perspective is essential because the feasibility of federated quantum learning is jointly constrained by data encoding, non-local circuit depth, entanglement availability, latency, and hardware coherence.

The remainder of the paper is organized as follows. Section 2 introduces the distributed distance-based classifier and its interpretation as a federated learning primitive. Section 3 describes the hybrid space–ground satellite architecture, including entanglement generation, multiplexing, latency, and memory effects. Section 4 presents the neutral-atom processor noise model, dataset preprocessing, and experimental design. Section 5 reports the simulation results across multiple coherence regimes, feature dimensions, shot budgets, and numbers of parties. Section 6 discusses the implications for scalable satellite-enabled federated quantum learning, and Section 7 concludes the paper.

## 2. Distributed Quantum Classification for Federated Learning

### 2.1. Distance-Based Quantum Classification

Distance-based learning models classify a query point by comparing it to labeled training points using a similarity or distance metric. In the quantum distance-based classifier of Schuld et al., this similarity is obtained through a quantum interference experiment after the data have been amplitude-encoded into quantum states [13]. The distributed formulation of Neumann and Wezeman extends this idea to the setting where different parties hold different subsets of the data and jointly contribute to the classification process [12].

Let $x\in R^{M}$ be a normalized real-valued feature vector. In amplitude encoding, x is mapped to the quantum state defined in Equation (1),

(1) $$|x\rangle \equiv \left|\psi (x)\rangle =\sum_{i=0}^{M-1}x_{i}\right|i\rangle ,$$

where ${∥x∥}_{2}=1$. The inner product between two encoded states being

(2) $$k(x_{1},x_{2})=\langle \psi \left(x_{1}\right)\psi \left(x_{2}\right)\rangle .$$

The kernel defined in Equation (2) acts as a similarity measure and may be estimated through a simple interference circuit [13]. For normalized real vectors, the squared Euclidean distance follows as follows:

(3) $${∥x_{1}-x_{2}∥}_{2}^{2}=2\left(1-\langle \psi (x_{1})\rangle \psi (x_{2})\right).$$

**Figure 1:**
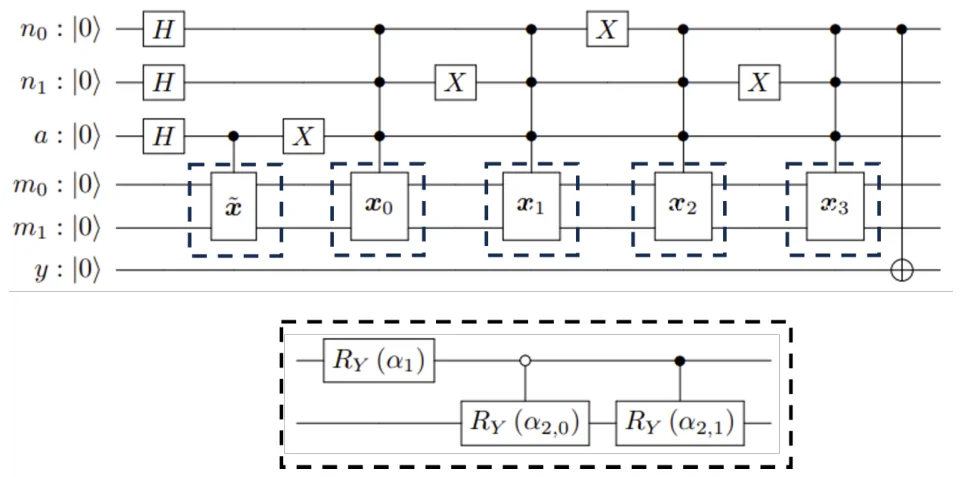
Example circuit of a distributed quantum distance-based classifier (adapted from [25]). The controlled blocks (in dashed boxes in the top circuit) represent data-encoding operations contributed by different parties. The inset (dashed box below the circuit) illustrates a state-preparation circuit for a two-feature vector using $R_{Y}$ and controlled-$R_{Y}$ rotations.

Given labeled training points ${\left\{\left(x_{i},y_{i}\right)\right\}}_{i=1}^{N}$ and a query point $\tilde{x}$, the distributed classifier computes

(4) $$\hat{y}=\mathrm{sgn}\left(\sum_{i=1}^{N}y_{i}\left(1-\frac{∥\tilde{x}-x_{i}{∥}_{2}^{2}}{4N}\right)\right),$$

where $y_{i}\in \left\{-1,+1\right\}$. The quantum subroutine is therefore used to estimate the overlaps or distances required by the decision function shown in Equation (4). A representative circuit implementation of the distributed quantum distance-based classifier is shown in Figure 1.

### 2.2. Distributing the Distance-Based Classifier

The quantum distance-based classifier (QDBC) estimates overlaps between amplitude-encoded quantum states and uses these overlaps to compute distances and perform classification [13]. In many practical applications, however, training data are distributed across multiple parties. A distributed QDBC seeks to preserve the overlap-estimation procedure of the centralized algorithm while avoiding direct sharing of raw data [12].

In the distributed quantum computing (DQC) model, several quantum processors are connected through quantum and classical communication channels. Local operations are performed within each node, while interactions between nodes are realized using shared entanglement, local operations, measurements, and classical feedforward [8,12]. This allows nonlocal controlled operations to be implemented without physically transferring data qubits.

The key challenge in distributed QDBC is the amplitude-encoding stage. The protocol of Neumann and Wezeman requires controlled state-preparation operations conditioned on server-held index and selector registers [12]. When the control qubit resides at the server and the target qubit is hosted by a remote party, the controlled rotation becomes a nonlocal operation.

**Figure 2:**
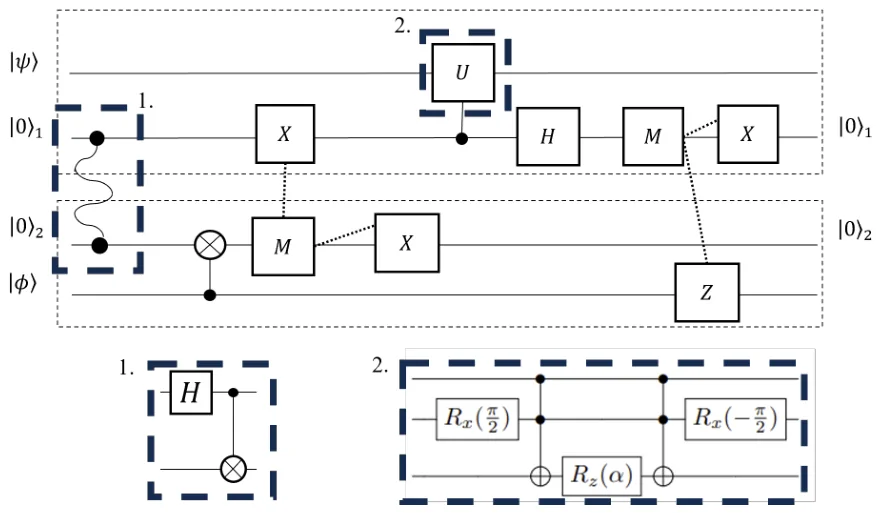
Distributed control primitive used to implement a nonlocal controlled operation between a server-held control qubit and a remotely hosted target qubit. Shared entanglement, local operations, measurements, and feedforward corrections enable the realization of a distributed controlled-$R_{Z}\left(\theta \right)$ gate, which forms the nonlocal component of the amplitude-encoding routine.

Since amplitude encoding is constructed from $R_{Y}$ and controlled-$R_{Y}$ rotations, we rewrite $R_{Y}$ according to Equation (5),

(5) $$R_{Y}\left(\theta \right)=R_{X}\;\left(\frac{\pi }{2}\right)R_{Z}\left(\theta \right)R_{X}\;\left(-\frac{\pi }{2}\right),$$

so that only the controlled-$R_{Z}\left(\theta \right)$ needs to be implemented nonlocally. The surrounding $R_{X}$ rotations are executed locally by the data-owning party. This reduces distributed state preparation to the realization of distributed controlled phase rotations, which can be implemented using cat-entangler/cat-disentangler or GHZ-assisted control primitives [8,12]. The corresponding distributed control primitive used throughout the protocol is illustrated in Figure 2.

Operationally, the server prepares the selector and index registers and distributes entanglement to the participating parties. Each party then executes the local portion of the amplitude-encoding circuit. Whenever a controlled rotation depends on a server-held control qubit, a distributed control protocol temporarily correlates a local communication qubit with the server control, applies the required controlled-$R_{Z}\left(\theta \right)$ operation locally, and subsequently removes the auxiliary correlations through measurements and feedforward corrections. After all distributed rotations have been executed, the global state is equivalent to that of the centralized classifier and can be used for the standard interference-based overlap estimation [12].

Once state preparation has been completed, the remainder of the classifier proceeds exactly as in the centralized setting. Interference measurements estimate the quantum kernel:

(6) $$k\left(\tilde{x},x_{i}\right)=\langle \psi \left(\tilde{x}\right)|\psi \left(x_{i}\right)\rangle ,$$

from which distances are computed from the query-training kernel in Equation (6) using Equation (3) and classification is performed [12,13]. In the healthcare setting considered here, each hospital acts as a data-owning party that prepares quantum states derived from local patient records, while a central server coordinates the protocol and performs the final inference. The protocol, therefore, enables collaborative analysis while limiting the exchange of raw patient data.

### 2.3. Prediction Error and Confidence Intervals

Even in the absence of hardware noise, quantum classifiers exhibit statistical uncertainty due to finite-shot sampling. This prediction error is distinct from decoherence or gate errors and reflects the uncertainty in estimating outcome probabilities from a limited number of measurements.

Let

(7) p=Pr(y=+1)

denote the ideal probability of predicting class +1. After executing the circuit for *N* shots and observing *K* positive outcomes, the probability is estimated by

(8) $$\hat{p}=\frac{K}{N}.$$

Assuming independent shots and using the ideal class probability defined in Equation (7), K∼Bin(N,p) and the estimator variance is

(9) $$\mathrm{Var}\left(\hat{p}\right)=\frac{p(1-p)}{N}.$$

Thus, the uncertainty decreases as $N^{-1/2}$. For sufficiently large *N*, an approximate (1−α) confidence interval is

(10) $$\hat{p}\pm z_{\alpha /2}\sqrt{\frac{\hat{p}\left(1-\hat{p}\right)}{N}},$$

where $z_{\alpha /2}$ is the corresponding standard-normal quantile. If this interval overlaps the decision boundary (p=1/2), the classification cannot be regarded as statistically significant at the chosen confidence level.

Prediction error therefore arises from finite sampling, whereas hardware noise changes the underlying probability *p*. Reporting confidence intervals alongside classification results provides a direct measure of inference reliability. The probability estimator of Equation (8), its variance in Equation (9), and the confidence interval of Equation (10) provide the statistical framework used to interpret shot-noise-induced uncertainty.

### 2.4. Implementation Details

We consider the distributed classifier of Neumann and Wezeman [12], where a server classifies a query point $\tilde{x}$ using data held by *K* parties. Each party contributes *N* normalized training samples with *M* features.

The dominant distributed operation is amplitude encoding. A length-*M* normalized vector can be prepared using a binary-tree state-preparation circuit containing (M−1) controlled rotations [12]. Since the global state incorporates all NK training samples, the leading entanglement-resource scaling is

(11) $$N_{\mathrm{GHZ}}∼N\;K\;\left(M-1\right).$$

Equation (11) highlights the linear dependence of the entanglement requirements on the number of parties, training samples, and encoded features.

Any controlled rotation whose control and target reside on different nodes must be implemented through shared entanglement and classical communication. We therefore parameterize the resource cost in Equation (12) as

(12) $$N_{\mathrm{GHZ}}=c_{\mathrm{ent}}\left(d\right)\;N\;K\;\left(M-1\right),$$

where $c_{\mathrm{ent}}\left(d\right)$ captures the fixed overhead associated with implementing a distributed controlled rotation and

$$d=2^{\left\lceil \log_{2}M\right\rceil }$$

is the Hilbert-space dimension used for amplitude encoding.

**Figure 3:**
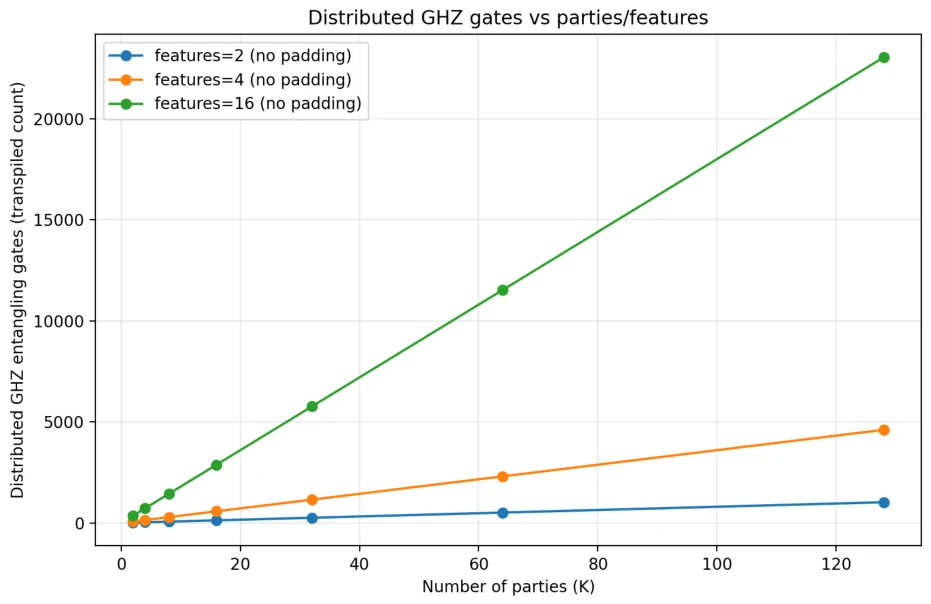
Scaling of distributed entanglement resources for the distributed classifier circuit. The number of GHZ-related entangling gates grows approximately linearly with the number of parties *K*, with a larger slope for higher feature dimensions.

Empirically, the compiled circuits exhibit

(13) $$N_{\mathrm{GHZ}}=\left\{\begin{array}{cc} 4\;N\;K\;(M-1), & d=2,\\ 6\;N\;K\;(M-1), & d\geq 4, \end{array}\right.$$

demonstrating the expected linear scaling with the number of parties, training samples, and encoding complexity. The measured scaling of the compiled circuits is shown in Figure 3. Equation (13) summarizes the experimentally observed coefficients used throughout this work.

## 3. Hybrid Space–Ground Satellite Architecture

### 3.1. System Concept

We consider a hybrid space–ground quantum network in which a satellite distributes entanglement between distant ground stations. Each ground station hosts a local quantum processor and the corresponding communication/memory components required to consume entanglement for distributed operations. The satellite does not perform the classification directly; rather, it provides the non-local entanglement resource that enables federated quantum learning across geographically distributed parties.

This architecture addresses the fundamental range limitation of terrestrial quantum links. While fiber-based quantum networks are suitable for short and medium distances, their performance degrades rapidly with distance due to optical loss. Satellite-based free-space links offer a complementary solution capable of supporting regional and intercontinental quantum connectivity [19].

### 3.2. Single-Satellite Entanglement Distribution

**Figure 4:**
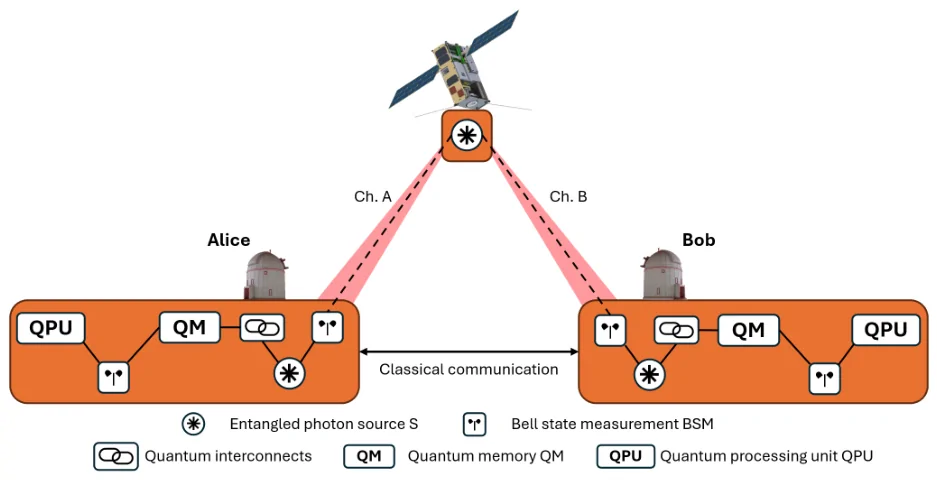
Single-satellite hybrid space–ground entanglement-distribution architecture. A satellite-hosted entangled photon source distributes photons to ground stations through free-space channels, while Bell-state measurements herald successful loading into quantum memories.

In the baseline architecture, the satellite hosts an entangled photon source and distributes one photon of each pair to each of two ground stations through free-space optical channels. At the ground segment, quantum memories store the photonic states and interface with the local quantum processing unit through quantum interconnects, allowing distributed entanglement resources to be transferred from the communication layer to the computational layer. Successful loading is heralded through Bell-state measurements. The considered single-satellite architecture is depicted in Figure 4.

The probability of entangling two quantum memories through a satellite-mediated link is modeled as [19,26]

(14) $$\begin{array}{cc} p_{e} & =\left(1-{\left(1-\eta_{ch:A}\eta_{ch:B}\eta_{sys}\right)}^{\Theta }\right)\eta_{QM}^{2},\\ \eta_{sys} & =\eta_{S}^{3}\eta_{BSM}^{2}\eta_{DET}^{4}, \end{array}$$

where $\eta_{ch:A}$ and $\eta_{ch:B}$ denote the channel transmission efficiencies, $\eta_{QM}$ is the quantum-memory loading efficiency, $\eta_{S}$ is the source efficiency, $\eta_{BSM}$ is the Bell-state measurement efficiency, $\eta_{DET}$ is the detector efficiency, and Θ is the number of multiplexed modes. Equation (14) forms the basis for the entanglement-generation model used throughout the communication-layer simulations. This expression accounts for the efficiencies associated with entanglement generation, transmission, detection, Bell-state measurements, multiplexing, and memory loading, and therefore isolates the dominant long-distance communication-layer contributions. A more complete hardware model should also account for the local quantum interconnect between the quantum memory and the local QPU, shown in Figure 4. We denote the corresponding memory-to-processor transfer efficiencies at the two ground stations by $\eta_{\mathrm{int}:A}$ and $\eta_{\mathrm{int}:B}$. Including these local interface losses modifies Equation (14) to

(15) $$p_{e}^{\left(\mathrm{int}\right)}=\left(1-{\left(1-\eta_{ch:A}\eta_{ch:B}\eta_{sys}\right)}^{\Theta }\right)\eta_{QM}^{2}\eta_{\mathrm{int}:A}\eta_{\mathrm{int}:B}.$$

For a symmetric local interface, $\eta_{\mathrm{int}:A}=\eta_{\mathrm{int}:B}=\eta_{\mathrm{int}}$, Equation (15) reduces to

(16) $$p_{e}^{\left(\mathrm{int}\right)}=p_{e}\eta_{\mathrm{int}}^{2}.$$

The interconnect loss therefore enters Equation (14) as a simple multiplicative correction. For instance, a local memory-QPU transfer efficiency in the range $\eta_{\mathrm{int}}\in \left[0.8,0.99\right]$ would reduce the usable entanglement probability by $\eta_{\mathrm{int}}^{2}\in \left[0.64,0.98\right]$ in the symmetric case, and would increase the expected number of attempts by approximately $1/\eta_{\mathrm{int}}^{2}$. In the simulations presented below, we set $\eta_{\mathrm{int}}=1$, corresponding to an ideal local memory-QPU interface. This is an optimistic assumption, but it keeps the focus on the satellite-mediated communication layer. Equations (15) and (16) show that finite interconnect efficiency mainly reduces the usable entanglement rate, without changing the qualitative scaling with channel loss, multiplexing, memory efficiency, or the number of distributed controlled operations. More broadly, the model highlights that successful entanglement distribution is probabilistic and depends not only on the satellite link, but also on memory loading, component efficiencies, and local memory-QPU transfer.

### 3.3. Inter-Satellite Links and Repeater-like Extension

**Figure 5:**
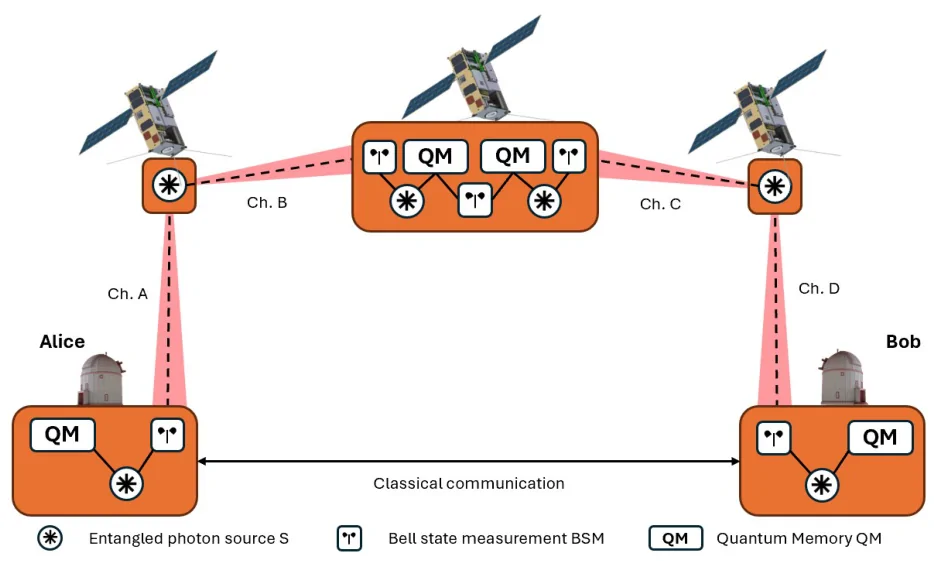
Inter-satellite extension of the hybrid quantum network architecture. Edge satellites generate entangled pairs, while an intermediate node performs Bell-state measurements and memory-assisted entanglement swapping.

For longer ranges or more challenging geometries, one may introduce inter-satellite links and entanglement swapping using quantum memories and Bell-state measurements [14,15]. In that case, elementary links are generated independently and subsequently connected to extend the total distance. A schematic illustration of this inter-satellite extension is provided in Figure 5.

Although the main results in this paper focus on the single-satellite case, the architecture and formulas naturally generalize to inter-satellite settings. This is relevant for the long-term scalability of federated quantum learning over global quantum networks.

### 3.4. Latency, Quantum Memories, and Execution Time

Distributed non-local gates are not only loss-limited but also latency-limited. The communication latency is estimated using Equation (17). If the distance between two parties is *D*, the communication round-trip time is approximated by

(17) $$t_{\mathrm{latency}}=\frac{2D}{c}.$$

This latency is the dominant timescale for distributed gate execution in the considered scenarios, since local one- and two-qubit gates are much faster than long-distance classical feed-forward [27].

In this work, the total duration of one classifier shot is approximated using a sequential latency model,

(18) $$\Delta T_{\mathrm{seq}}=N_{GHZ}t_{latency},$$

where $N_{GHZ}$ is the number of non-local entanglement-consuming operations. Equation (18) assumes that these operations are executed one after another, so it should be interpreted as a conservative estimate of the shot duration. In a more optimized implementation, entanglement generation could be partially overlapped through pipelining or spatial multiplexing. If $P_{\mathrm{eff}}$ usable entangled resources can be prepared per latency window, the effective duration would instead scale as

(19) $$\Delta T_{\mathrm{pipe}}≃\left\lceil \frac{N_{GHZ}}{P_{\mathrm{eff}}}\right\rceil t_{latency}.$$

The sequential model in Equation (18) corresponds to $P_{\mathrm{eff}}=1$. In the simulations below, we use Equation (18). Possible latency hiding through pipelining is therefore not explicitly exploited. Quantum memories remain necessary in both cases, since entangled pairs prepared before use must be stored and are exposed to memory dephasing.

## 4. Materials and Methods

### 4.1. Noise-Aware Neutral-Atom Processor Model

The quantum processors are modeled as neutral-atom devices based on Rydberg-mediated entangling gates, where qubits are encoded in long-lived hyperfine ground states and interactions are generated through transient excitation to Rydberg levels [20,21]. This platform combines strong optical connectivity with high-fidelity entangling operations, with recent experiments reporting two-qubit gate fidelities approaching 99.5% [22,28]. However, the dominant error mechanisms differ from those in superconducting or photonic architectures and include spontaneous Rydberg decay, off-resonant scattering, laser phase noise, and electric-field-induced dephasing [29,30].

To capture these effects at the circuit level, we employ a time-aware CPTP noise model. Quantum channels are represented using the Kraus formalism in Equation (20),

(20) $$E\left(\rho \right)=\sum_{k}K_{k}\rho K_{k}^{†},\;\sum_{k}K_{k}^{†}K_{k}=I,$$

which guarantees physically valid open-system evolution [31].

Decoherence during both gate execution and idle periods is modeled through amplitude damping and phase damping channels parameterized by the operation duration Δt, the relaxation time $T_{1}$, and the coherence time $T_{2}$. Following standard circuit-level noise models, the relaxation channel is written as Equation (21),

(21) $$E_{\mathrm{relax}}=E_{\mathrm{deph}}\left(T_{\phi },\Delta t\right)\circ E_{\mathrm{AD}}\left(T_{1},\Delta t\right),$$

where the pure-dephasing time $T_{\phi }$ is related to $T_{1}$ and $T_{2}$ through Equation (22),

(22) $$\frac{1}{T_{2}}=\frac{1}{2T_{1}}+\frac{1}{T_{\phi }},$$

and the corresponding damping probabilities scale with the physical duration of the instruction according to Equation (23), as

(23) $$\gamma_{1}=1-e^{-\Delta t/T_{1}},\;\gamma_{\phi }=1-e^{-\Delta t/T_{\phi }}.$$

This allows decoherence to accumulate continuously over the actual time budget of the circuit, including idle qubits [32,33].

Single-qubit control errors are represented by an effective depolarizing channel in Equation (24), calibrated to the experimentally reported average single-qubit gate fidelity $F_{1q}$,

(24) $$E_{1q}\left(\rho \right)=\left(1-p_{1}\right)\rho +p_{1}\frac{I}{2},\;p_{1}=2\left(1-F_{1q}\right).$$

For two-qubit Rydberg gates, the intended unitary operation is followed by an effective noise channel as defined in Equation (25),

(25) $${\tilde{U}}_{2q}\left(\rho \right)=E_{\mathrm{Ry}}^{\left(2\right)}\;\left(U_{2q}\rho U_{2q}^{†}\right),$$

which captures spontaneous Rydberg decay, dephasing, and residual stochastic processes such as off-resonant scattering and imperfect blockade. The overall error rate is calibrated to the reported two-qubit gate fidelity $F_{2q}$ through the effective depolarizing probability in Equation (26),

(26) $$p_{2}=\frac{4}{3}\left(1-F_{2q}\right),$$

with the error budget partitioned among the dominant physical mechanisms.

Finally, measurement noise is modeled as a classical assignment-error process corresponding to state-dependent fluorescence detection. Readout errors are described by a symmetric confusion matrix in Equation (27),

(27) $$P\left(m|b\right)=\left(\begin{array}{cc} 1-p_{m} & p_{m}\\ p_{m} & 1-p_{m} \end{array}\right),$$

where $p_{m}$ is chosen to reproduce the experimentally reported SPAM fidelity [22,28].

The resulting framework captures the principal physical error channels of contemporary neutral-atom quantum processors while remaining compatible with efficient circuit-level simulation and distributed quantum classification studies.

### 4.2. Dataset and Federated Learning Interpretation

As a representative privacy-sensitive task, we use the Breast Cancer Wisconsin (Diagnostic) dataset from the UCI Machine Learning Repository [23,24]. The dataset contains 569 labeled patient cases and 30 real-valued features extracted from digitized fine-needle aspirate images. In this work, the dataset is used as a stand-in for a federated medical classification task in which multiple hospitals wish to collaboratively classify data without sharing their raw patient records.

The dataset is partitioned horizontally across *K* parties. Each party contributes a subset of labeled training points, while a central server coordinates the distributed classification of a query point. Before amplitude encoding, the selected features are standardized and normalized. Because amplitude encoding naturally maps vectors of dimension $2^{m}$ onto *m* qubits, we restrict attention to feature subsets whose dimension is a power of two.

### 4.3. Experimental Design

The experimental campaign varies four main parameters, namely, the number of parties, feature dimensions, shot count, and coherence scenarios. These parameter configurations are summarized in Table 1.

The feature subsets are the following: for the two-feature case we use concave_points_worst and radius_worst, while for the four-feature case, we extend this set with area_worst and concave_points_mean. The selected features were chosen because they provide meaningful low-dimensional classification structure, while still permitting amplitude encoding within a feasible circuit size.

The distributed network scenarios are mapped to realistic geographic configurations, ranging from a two-party European setting to larger multi-party configurations, including intercontinental links. The complete set of ground-station locations considered across the experimental campaign consists of Amsterdam, Berlin, Lisbon, New York, Rotterdam, Málaga, Rome, and Bucharest. The satellite used for conducting the experiments is a medium earth orbit (i.e., MEO) NAVSTAR configuration at a zenith angle of 70 degrees.

Each set of experiments was executed on the TNO HPC environment with a maximum wall-clock allocation of seven days per run. Each task was run as a single SLURM array job using one process, 4 CPU cores, and 16 GB of memory to support parallelized simulations. All configurations were executed within a Python 3.12 virtual environment. Circuit-level simulations were performed using the AerSimulator from Qiskit Aer configured with the Matrix Product State (MPS) simulation method. The MPS representation expresses the quantum state as a one-dimensional tensor network and is particularly suitable for simulating circuits whose entanglement structure remains limited, although its computational cost increases with the amount of entanglement generated during circuit evolution [34,35]. MPS-based tensor-network structures have also been investigated as quantum classifiers for classical and quantum data [36], making this formalism naturally connected to the quantum-classification setting considered here.

### 4.4. Evaluation Metrics

For each experiment, we report:

1. the predicted class label and whether it matches the ground truth,
2. the class vote counts $C_{0}$ and $C_{1}$ after post-selection,
3. the decision margin
  (28) $$\Delta =\frac{|C_{0}-C_{1}|}{N_{\mathrm{shots}}},$$
4. the wall-clock execution time of the classical simulation,
5. circuit-level resource metrics such as qubit count and transpiled depth.

The decision margin defined in Equation (28) serves as a compact indicator of confidence: values close to zero indicate effectively random classification, while large values indicate a strong class preference. In addition, the shot-based interpretation of the classifier permits a binomial confidence-interval view on the prediction uncertainty, separating finite-shot uncertainty from hardware-induced algorithmic degradation.

## 5. Results

### 5.1. Overview

Across the full experimental campaign, the classifier exhibits two distinct performance regimes. In low-coherence scenarios, the classification outcomes are either effectively random or strongly biased by decoherence. In the high-coherence scenario, the classifier behaves correctly for the two-feature case but still fails in the four-feature case due to limitations of the classical feature representation rather than the quantum hardware. This separation is one of the main results of the paper.

For Scenarios 1-3, all 16 experimental configurations completed successfully, allowing the full range of party counts (K=2,3,4,8) to be analyzed. In Scenario 4, however, the substantially increased cost of classical simulation prevented three of the four K=8 configurations from completing within the available wall-clock limit. Consequently, only 13 of the 16 planned experiments finished successfully. For Scenarios 1 and 3, the figures include the K=2 and K=8 cases with both two-feature and four-feature encodings. For Scenario 4, where only a single K=8 experiment completed, the figures instead show the K=2 and K=4 cases.

Furthermore, the full results obtained in this study can be seen in Appendix A. Figure A1, Figure A2, Figure A3, Figure A4, Figure A5, Figure A6, Figure A7, Figure A8, Figure A9, Figure A10, Figure A11, Figure A12, Figure A13, Figure A14, Figure A15 and Figure A16 show the classification outcomes for all completed experiments. Table A1, Table A3, Table A5 and Table A7 display the classification outcomes for scenarios 1 through 4, while Table A2, Table A4, Table A6 and Table A8 portray the wall-clock execution time on the HPC cluster for each scenario.

### 5.2. Near-Term Coherence Regimes: Decoherence-Dominated Behavior

In Scenarios 1 and 2, the two-feature experiments produce decision margins near zero across party counts, indicating that the classifier behaves almost randomly. This means that nominally correct labels in these regimes should not be interpreted as reliable classification, but rather as outcomes produced without robust discriminatory power.

The four-feature experiments under the same coherence conditions behave differently: instead of fluctuating around a balanced result, they collapse systematically toward one output class. This indicates that the deeper distributed circuits associated with the four-feature encoding are strongly affected by decoherence, causing the quantum state to degrade toward a biased output.

Across all experiments, Scenarios 1 and 2 exhibit the same qualitative behavior. The two-feature experiments produce decision margins in the ranges Δ∈[0.000,0.062] and Δ∈[0.002,0.026], respectively, whereas the four-feature experiments exhibit a systematic class-0 collapse with margins of Δ∈[0.850,0.977] and Δ∈[0.860,0.900].

**Figure 6:**
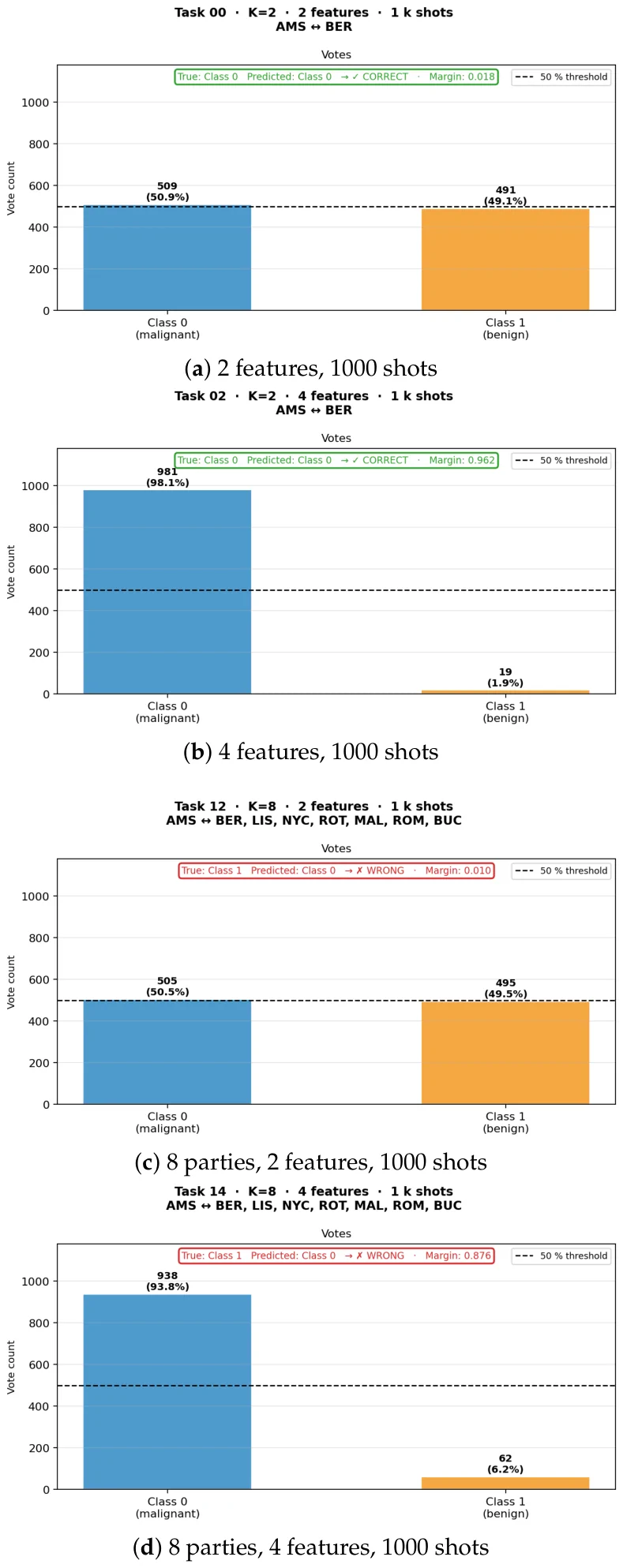
Results for Scenario 1 ($T_{1}=100$ µs, $T_{2}=50$ µs), illustrating near-random outcomes for the two-feature case and strong class collapse for the four-feature case.

Representative results for Scenario 1 are shown in Figure 6. Scenario 2 exhibited the same qualitative behavior as Scenario 1 and is therefore omitted from the main figures for brevity. The full results are provided in Appendix A.

### 5.3. Intermediate Regime: Partial Transition

**Figure 7:**
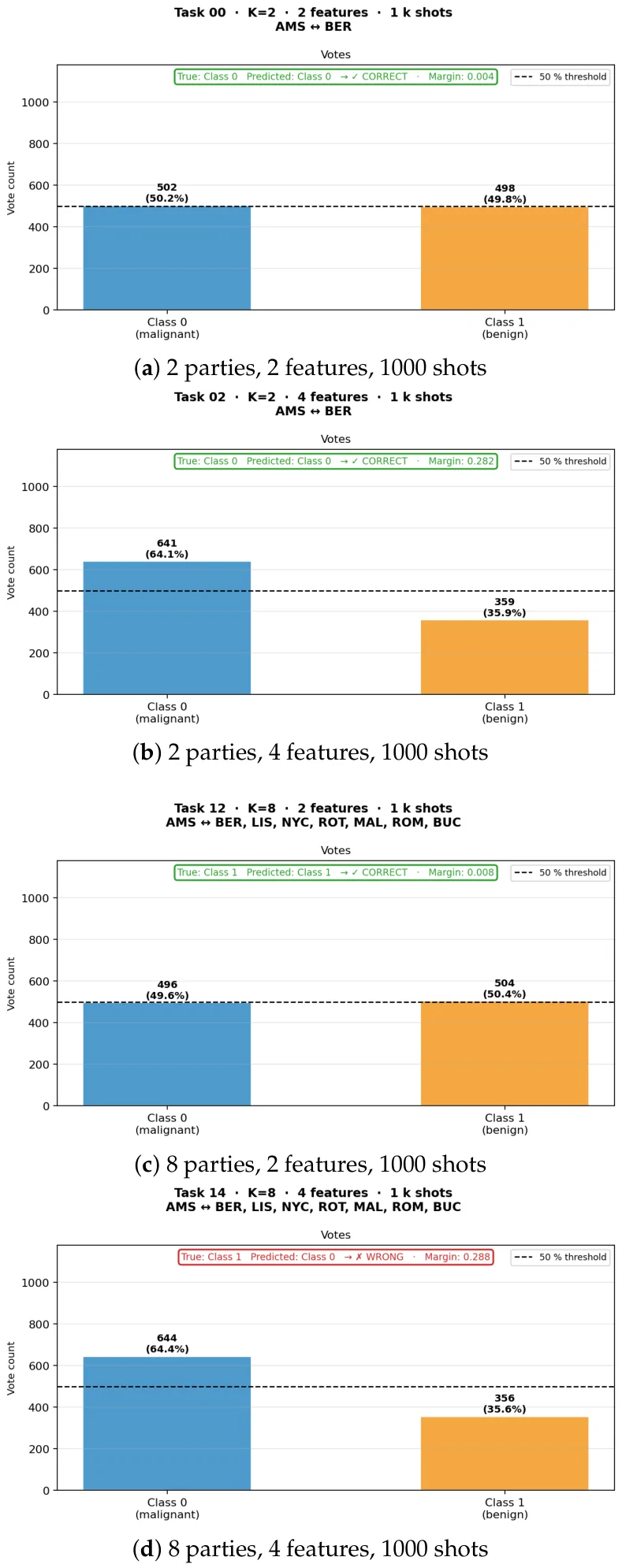
Results for Scenario 3 ($T_{1}=80\;\mathrm{ms}$, $T_{2}=40\;\mathrm{ms}$). Performance on the two-feature task remains at a near random level, while the class collapse for the four-feature task becomes less severe.

Scenario 3 ($T_{1}$ = 80 ms; $T_{2}$ = 40 ms) displays a transition behavior. The two-feature experiments are still near-random, but the four-feature class collapse becomes visibly less extreme than in Scenarios 1 and 2. This suggests that the classifier lies near a hardware threshold: coherence is no longer so poor that the circuit fully collapses immediately, but it is still insufficient for reliable algorithmic discrimination. This behavior can be observed in Figure 7.

The observed reduction in class-collapse behavior relative to Scenarios 1 and 2 suggests the presence of an intermediate operating regime between strong decoherence and reliable classification.

### 5.4. High-Coherence Regime: Algorithm Succeeds, but Data Representation Fails

The most informative behavior appears in Scenario 4 ($T_{1}$ = 10 s, $T_{2}$ = 1 s). For the two-feature setting, the classifier yields correct predictions with large decision margins across all completed experiments. In other words, once sufficient coherence is available, the distributed classifier can survive the circuit depth and network overhead imposed by the satellite-enabled architecture.

However, the four-feature experiments remain poor. In this regime, the outputs are no longer strongly biased by decoherence; instead, they exhibit low margins and systematically incorrect behavior. Unlike the lower-coherence scenarios, the four-feature experiments no longer exhibit strong class collapse. Instead, they produce systematically incorrect predictions with comparatively small decision margins.

**Figure 8:**
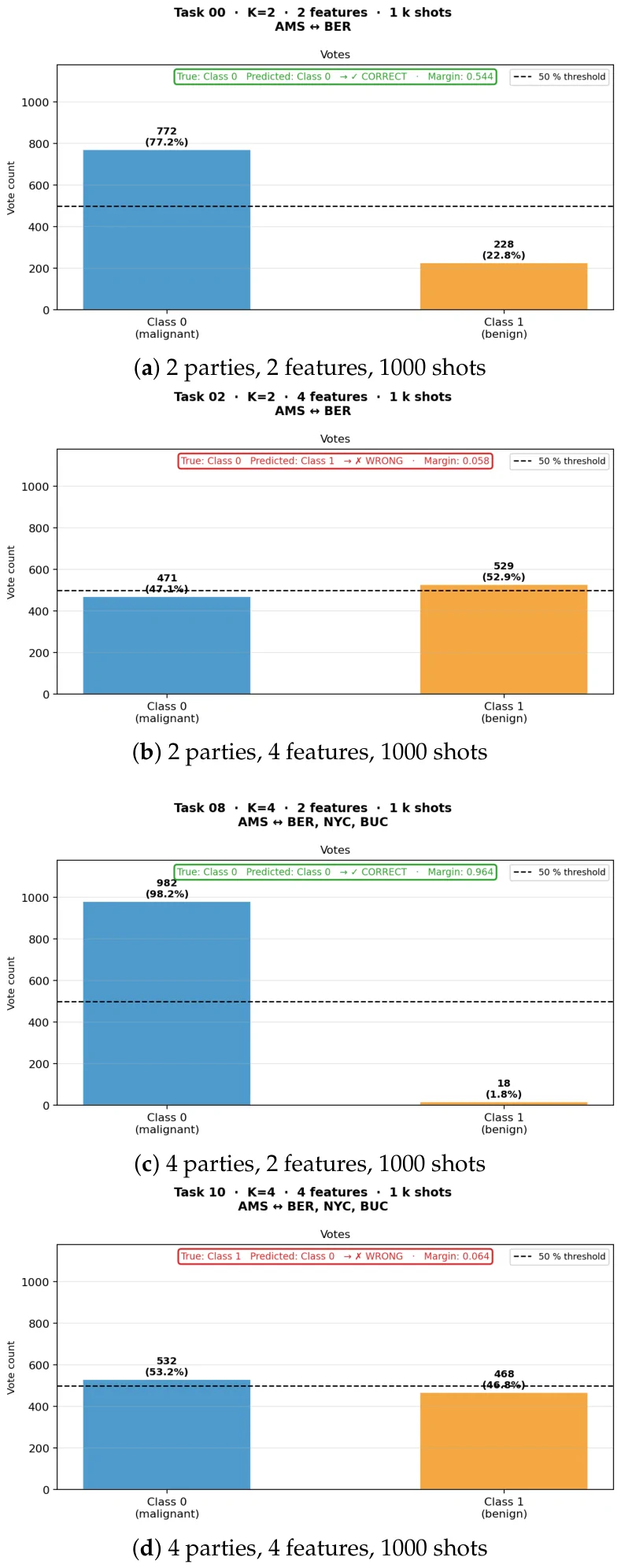
Representative Scenario 4 results ($T_{1}=10\;s$, $T_{2}=1\;s$). The two-feature task is classified correctly with large margins, whereas the four-feature task remains unreliable.

The high-coherence results are shown in Figure 8.

### 5.5. Classification Outcomes

Table 2 summarizes the numerical outcomes for the experiments shown in the main figures. In Scenario 1, the two-feature experiments produce nearly balanced vote distributions, with decision margins of only Δ=0.018 for K=2 and Δ=0.010 for K=8. In contrast, the four-feature experiments exhibit a strong collapse toward class 0, with margins of Δ=0.962 and Δ=0.876, respectively.

Scenario 3 displays intermediate behavior. The two-feature margins remain close to zero (Δ=0.004 and Δ=0.008), whereas the four-feature margins decrease substantially relative to Scenario 1, reaching Δ=0.282 and Δ=0.288. This indicates that the class-collapse behavior is weakened but not fully removed.

In Scenario 4, the two-feature classifier produces correct predictions with substantially larger decision margins, increasing to Δ=0.544 for K=2 and Δ=0.964 for K=4. The corresponding four-feature experiments remain unsuccessful, but unlike the earlier scenarios, they exhibit low margins (Δ=0.058 and Δ=0.064), suggesting that the dominant limitation is no longer decoherence but the feature representation itself.

Table 3 provides a performance aggregate for the classifier for the results shown throughout the section.

The aggregate results reveal a clear separation between low and high coherence regimes. While Scenarios 1–3 exhibit poor classification performance across both feature configurations, Scenario 4 achieves correct predictions for all completed two-feature experiments. In contrast, the four-feature configuration remains unsuccessful even in the highest-coherence regime. This transition is reflected in the decision margins of the two-feature classifier, which increase from approximately 0.01 in Scenario 1 to values between 0.54 and 0.96 in Scenario 4.

### 5.6. Simulation Costs

Table 4 showcases the simulation costs and circuit resources associated with running the experimental configurations mentioned in Section 4.3. The required resources grow substantially with both the number of parties and the number of features. For example, increasing the network size from K=2 to K=8 increases the qubit count from 13 to 28 for the two-feature configuration and increases the transpiled circuit depth from approximately $2.4\times 10^{3}$ to $6.7\times 10^{4}$ gates.

The simulation cost also depends strongly on the coherence regime. In Scenarios 1 and 3, all experiments are complete within approximately 15 min despite the large circuit depths. By contrast, the high-coherence regime of Scenario 4 is considerably more expensive to simulate, with the K=4 experiments requiring up to 1.3 h. This trend is consistent with the full experimental campaign, where the sole completed K=8 two-feature simulation in Scenario 4 required 89.1 h (see Table A8) and the remaining three K=8 configurations did not finish within the available wall-clock limit.

### 5.7. Feature-Space Conditioning & Nearest-Centroid Comparison

**Figure 9:**
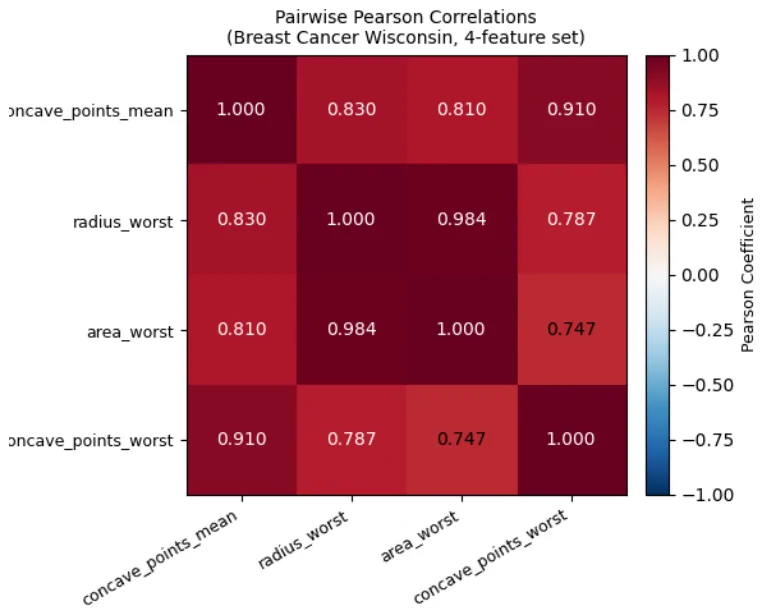
Pairwise Pearson correlation matrix for the four selected features used in the four-feature classifier experiments. Strong collinearity between radius_worst and area_worst contributes to the data-limited failure mode observed in the high-coherence regime.

To investigate the persistent four-feature failure observed in Scenario 4, we analyzed correlations within the selected feature subset. Figure 9 shows that radius_worst and area_worst are highly correlated, with a Pearson correlation coefficient of approximately 0.984.

The presence of this strong collinearity suggests that the four-feature experiments may be influenced not only by hardware effects but also by limitations of the selected feature representation. The implications of this observation are discussed further in Section 6 and Section 6.4.

**Figure 10:**
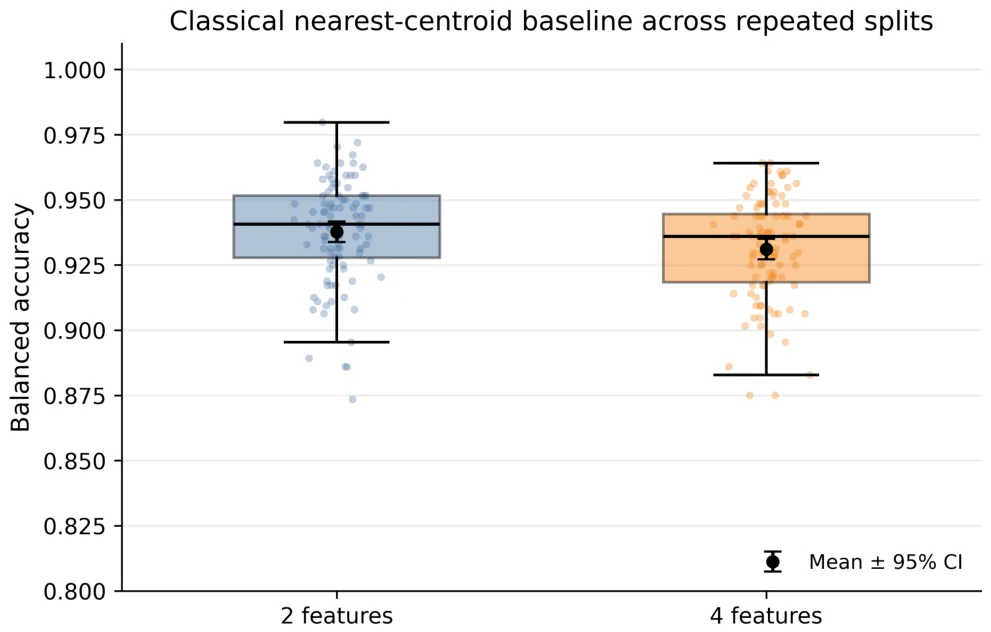
Balanced accuracy obtained by a classical nearest-centroid classifier across repeated train-test splits. The two-feature configuration performs comparably to, and slightly better than, the four-feature configuration, indicating that the additional features provide limited discriminative benefit.

To determine whether the observed four-feature behavior originates from the distributed quantum implementation or from the geometry of the selected feature space itself, we additionally evaluated a classical nearest-centroid classifier using the same feature subsets employed throughout this study. The nearest-centroid model was selected because it is fundamentally distance-based and therefore provides a natural classical analogue to the distance calculations performed by the distributed quantum classifier. Figure 10 summarizes the balanced classification accuracy obtained across repeated train-test splits of the Breast Cancer Wisconsin dataset. The two-feature configuration achieved a mean balanced accuracy of approximately 0.94, while the four-feature configuration achieved a slightly lower mean balanced accuracy of approximately 0.93. The overlap between the distributions indicates that introducing the additional dimensions does not provide a meaningful improvement in predictive performance. Instead, a small degradation in both accuracy and stability is observed.

**Figure 11:**
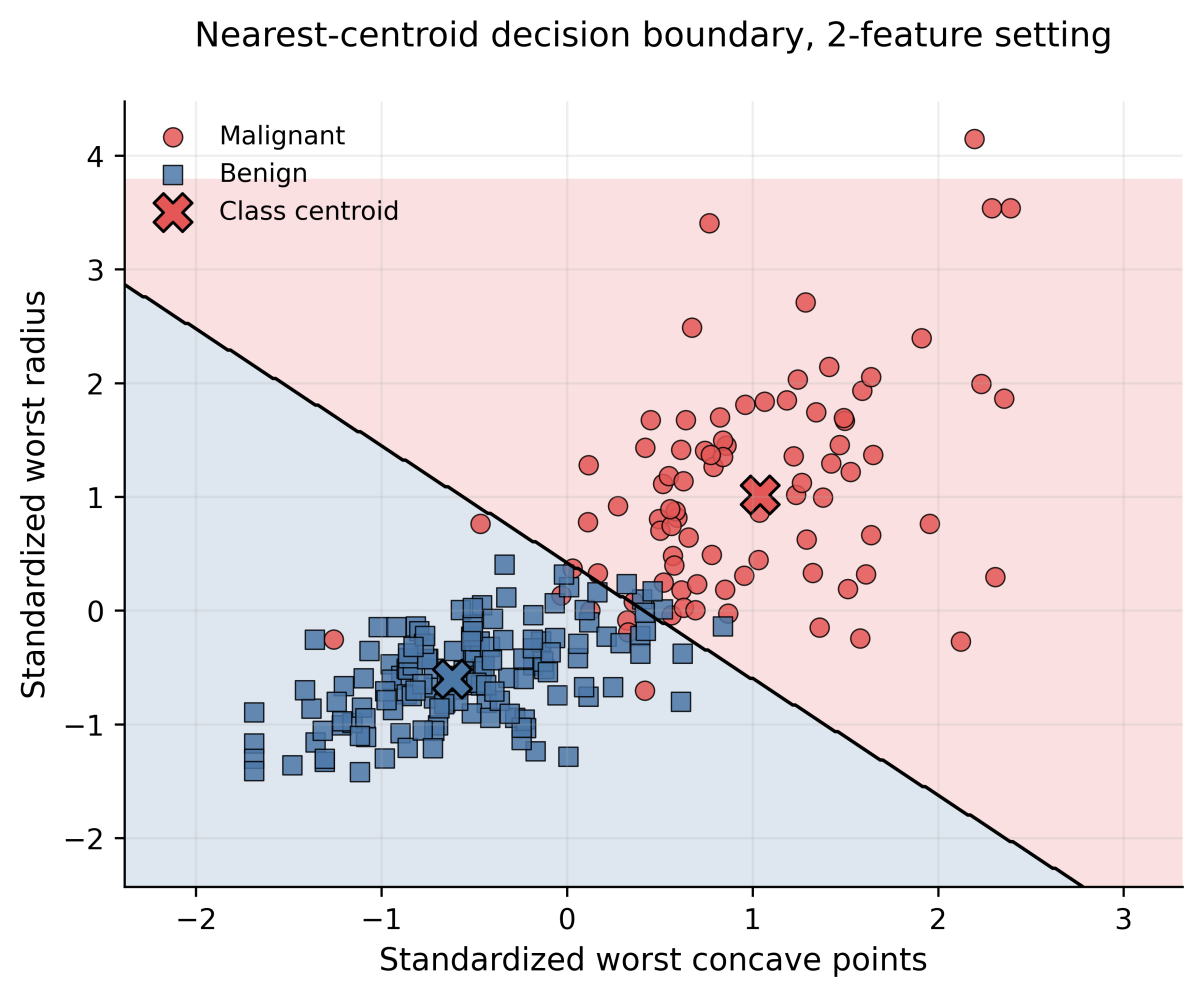
Decision boundary of the classical nearest-centroid classifier for the two-feature configuration. The centroid locations and resulting separation illustrate that the selected low-dimensional feature subset remains highly discriminative.

The decision boundary for the two-feature classifier is shown in Figure 11. The classes are well separated in feature space, with the malignant and benign centroids occupying distinct regions of the plane. The resulting decision surface achieves a balanced accuracy above 0.91, demonstrating that these two selected features already capture most of the class-discriminative information present in the dataset.

**Figure 12:**
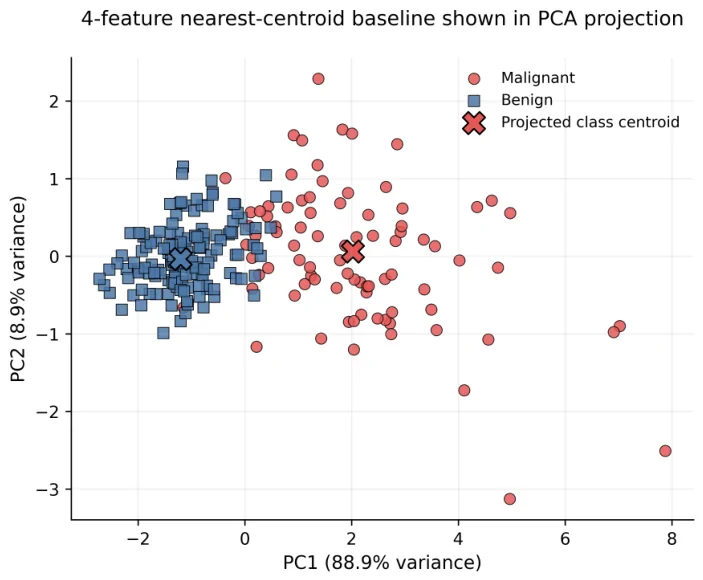
PCA projection of the four-feature nearest-centroid classifier. Although the classifier is trained in four dimensions, the additional features provide only limited improvement in class separation due to the strong redundancy present in the selected feature subset.

A projection of the four-feature classifier is shown in Figure 12. Although the classifier is trained in the full four-dimensional feature space, the PCA visualization reveals that the additional dimensions primarily alter the geometry of the feature distribution without substantially increasing class separation. This behavior is consistent with the correlation analysis shown in Figure 9, where radius_worst and area_worst exhibit near-perfect collinearity.

Taken together, the nearest-centroid results suggest that increasing the dimensionality of the selected feature subset does not necessarily improve classification performance. The additional features contribute information that is largely redundant with the original feature set, thereby increasing dimensionality without significantly increasing separability. This observation is consistent with the well-known concentration-of-distances phenomenon, whereby distance-based classifiers become progressively less sensitive to meaningful distinctions as redundant dimensions are added. Importantly, the nearest-centroid benchmark demonstrates that the four-feature representation remains classifiable with a balanced accuracy above 90%. Consequently, the complete failure observed for the distributed quantum classifier in Scenario 4 cannot be explained solely by the choice of features. To understand this, it is important to note that the distributed quantum classifier does not operate directly on Euclidean distances in the original feature space. Instead, each normalized feature vector x is amplitude encoded into a quantum state,

(29) $$|\psi \left(x\right)\rangle =\sum_{i=0}^{M-1}x_{i}|i\rangle ,$$

such that classification is based on overlaps of the form

(30) $$k\left(x_{i},x_{j}\right)=\langle \psi \left(x_{i}\right)|\psi \left(x_{j}\right)\rangle .$$

For normalized data, the overlap corresponds to the cosine of the angle between feature vectors rather than their Euclidean separation. As a result, highly correlated dimensions contribute nearly identical directions in Hilbert space. After *z*-score standardization and subsequent $\ell_{2}$ normalization, vectors that differ substantially in magnitude may become nearly parallel. The corresponding kernel values,

(31) $$k\left(x_{i},x_{j}\right)=x_{i}^{T}x_{j},$$

therefore become increasingly similar for samples belonging to different classes. The distributed classifier aggregates these overlaps according to

(32) $$\hat{y}=\mathrm{sgn}\left(\sum_{i}y_{i}\;k\left(x,x_{i}\right)\right),$$

where $y_{i}\in \left\{-1,+1\right\}$ are the training labels. When redundant dimensions compress the overlap distribution, positive and negative class contributions become more similar and increasingly cancel each other. In this regime, the decision score approaches zero even though the underlying classes remain separable under conventional distance-based classification. Therefore, the nearest-centroid baseline indicates that the four-feature failure observed in Scenario 4 is not solely a consequence of poor feature selection. Rather, it appears to arise from a combination of feature-space conditioning and the particular geometry induced by amplitude encoding and overlap-based classification. This observation suggests that feature sets that remain effective for classical distance-based methods may nevertheless become poorly conditioned when represented through normalized quantum amplitudes.

## 6. Discussion

### 6.1. Two Distinct Failure Modes

One of the main scientific findings of this work is the existence of two distinct failure modes. The first is a hardware-limited failure mode. In low-coherence regimes, the distributed circuit accumulates enough decoherence to destroy the class-discriminative quantum state. This manifests either as effectively random outcomes (two-feature case) or as a strong bias toward one output class (four-feature case).

The distinction between these behaviors is likely related to circuit complexity. The four-feature encoding requires substantially more distributed operations than the two-feature encoding, leading to greater cumulative exposure to decoherence. Consequently, the four-feature circuits exhibit a pronounced class-collapse behavior, whereas the two-feature circuits retain a sufficiently balanced output distribution to appear effectively random.

In this regime, the algorithm fails because the underlying computation is not preserved. The intermediate behavior observed in Scenario 3 further suggests that the transition between unusable and useful distributed classification is not governed by a single threshold. Instead, different error signatures emerge in different regions of parameter space, producing a distinct transition regime between strong decoherence and reliable operation.

The second is a *data-limited failure mode*. In the high-coherence regime, the two-feature classifier becomes reliable, showing that the architecture can support correct distributed inner-product estimation. Yet, the four-feature classifier still fails, now because the selected features are too collinear and poorly conditioned in the normalized inner-product space. In this regime, the algorithm fails not because the hardware is insufficient, but because the chosen data representation is not informative for the task. More broadly, this behavior is consistent with known limitations of distance-based learning methods in the presence of redundant or highly correlated features. As feature redundancy increases, the contrast between pairwise distances and inner products can diminish, reducing the ability of similarity-based classifiers to distinguish between classes. In the present case, the strong correlation between radius_worst and area_worst appears to distort the geometry of the normalized feature space, resulting in poor class separation despite successful quantum state preservation.

While the observed failure is strongly correlated with the collinearity of the selected feature subset, additional investigations using alternative feature-selection strategies would be required to determine the extent to which this behavior generalizes beyond the specific configuration studied here. Consequently, the present results should be interpreted as evidence of a data-limited failure mode for the selected feature representation rather than for all possible feature encodings of the dataset.

This distinction is important: improving the hardware alone does not solve all performance problems. Once the system crosses a hardware threshold, the classical structure of the encoded data becomes the dominant limitation.

### 6.2. Implications for Federated Quantum Machine Learning

From the application perspective, the results support the view that satellite-enabled federated quantum learning is feasible in principle. The DDBC demonstrates how multiple parties can contribute to a classification task through distributed quantum operations while retaining local control over their data. The satellite network architecture then extends this concept to long-distance settings, including intercity and intercontinental deployment scenarios.

At the same time, the study makes clear that this application is constrained by the joint behavior of communication, computation, and data representation. Practical feasibility therefore requires:

1. sufficiently high coherence times and gate fidelities to preserve the distributed circuit,
2. sufficiently strong entanglement generation and quantum-memory performance to support repeated non-local operations,
3. feature representations that are well conditioned under amplitude encoding and inner-product-based classification.

The path toward federated quantum machine learning is thus not only an algorithm design problem and not only a networking problem; it is inherently a systems integration problem.

### 6.3. Simulation Cost and Scalability

A further practical result concerns simulation complexity. The high-coherence scenarios are far more expensive to simulate classically than the low-coherence ones, because the Matrix Product State (MPS) backend scales with the amount of entanglement preserved during the simulation. This is visible in the high-coherence results: the K=4, two-feature, 1000-shot run required 50.6 min, while the corresponding K=8 run required 89.1 h. The remaining K=8 high-coherence configurations did not complete within the available wall-clock limit. These results should therefore be interpreted as a demonstration of small-scale feasibility under the simulated conditions, complemented by resource-scaling estimates for larger networks. They do not constitute a full numerical demonstration of large-scale high-coherence operation.

To make the scalability implications more quantitative, we estimate the resources required for larger networks using the circuit construction employed in this work. Let *p* denote the number of training points held per party per class, and let

(33) $$N_{\mathrm{pad}}=2^{\left\lceil \log_{2}\left(Kp\right)\right\rceil }$$

be the padded per-class training-set size. The corresponding qubit count is

(34) $$Q=2K+\log_{2}N_{\mathrm{pad}}+\log_{2}M+4,$$

while the number of entanglement-consuming distributed operations per shot is

(35) $$E=\left\{\begin{array}{cc} 4N_{\mathrm{pad}}, & M=2,\\ 6N_{\mathrm{pad}}\left(M-1\right), & M\geq 4. \end{array}\right.$$

In Equation (34), the first term accounts for one data qubit and one GHZ qubit per party, while the remaining terms correspond to the padded index register, amplitude-encoding register, label qubit, interference ancilla, and server-side GHZ qubit. Equation (35) gives the entangled-resource cost per classifier shot.

Table 5 applies Equations (33)–(35) to larger networks, fixing M=4, p=10 training points per party per class, 10,000 shots per query, and a 20 ms latency per non-local operation. The estimates are not additional simulations, but order-of-magnitude projections based on the same circuit construction.

These estimates show that quantum memory and communication requirements become the main scalability bottlenecks. A fully sequential implementation requires only a small number of entangled pairs to be stored at once, but the execution time grows directly with the number of distributed operations in Equation (35). Conversely, pipelining can reduce the latency, but only if enough quantum-memory modes are available to buffer many entangled pairs before they are consumed.

The classical communication overhead is comparatively modest. Each distributed operation exchanges two classical bits, so a full query requires 2E classical bits per shot count. Even for K=128 in Table 5, this corresponds to less than 100 MB of classical traffic for a $10^{4}$-shot query. The entangled-resource rate is more demanding: sustaining one such query per hour would require approximately $6\times 10^{3}$ usable GHZ pairs per second at K=8, increasing to about $10^{5}$ pairs per second at K=128.

The MPS memory estimates in Table 5 assume a maximum bond dimension χ=128, for which the storage cost is approximately $32Q\chi^{2}$ bytes in double precision. This remains far below the cost of a dense statevector, but only as long as the required bond dimension stays within the chosen cap. In the high-coherence regime, where more entanglement is preserved, faithful classical simulation becomes much more expensive; this is why the K=8 high-coherence case already required 89.1 h and why larger high-coherence networks become increasingly difficult to explore classically.

### 6.4. Limitations

Several limitations of this study should be acknowledged.

First, the experimental campaign evaluates the behavior of the distributed classifier using a small number of classification queries rather than a comprehensive held-out test-set evaluation. Consequently, the reported decision margins and classification outcomes should be interpreted primarily as indicators of algorithmic behavior under realistic communication and hardware constraints rather than as estimates of generalization performance on the full dataset. A statistically rigorous assessment of predictive accuracy would require repeating the complete distributed classification procedure for a large set of test instances. However, the computational cost of the distributed simulations grows rapidly with the number of parties, feature dimensions, and coherence times, making such an evaluation impractical within the available simulation budget.

Second, the four-feature configuration used in this study was selected primarily to investigate the effect of increased circuit complexity and resource consumption, rather than to maximize class separability in the amplitude-encoded feature space. Subsequent analysis revealed that two of the selected features, radius_worst and area_worst, exhibit near-perfect collinearity within the dataset (see Figure 9). After standardization and normalization, this strong correlation reduces geometric diversity in the amplitude-encoded states and degrades the discriminative power of inner-product-based classification. Consequently, the poor four-feature performance observed in Scenario 4 should not be interpreted as evidence that the distance-based classifier necessarily fails for higher-dimensional feature representations. Rather, it reflects the limitations of the specific feature subset investigated here. Additional studies employing alternative feature-selection and dimensionality-reduction strategies would be required to determine the extent to which the observed data-limited failure mode generalizes beyond the present setup.

**Figure A16:**
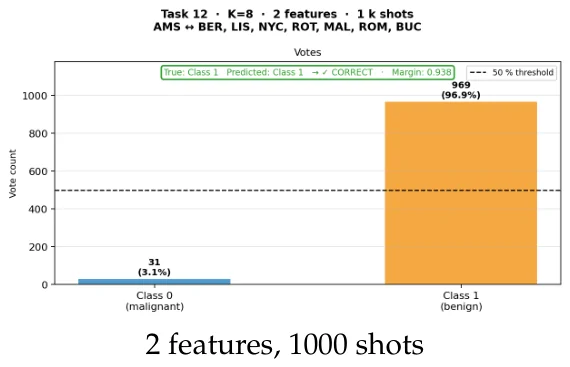
Results for K=8 parties (Amsterdam, Berlin, Lisbon, New York, Rotterdam, Málaga, Rome, Bucharest).

Third, the high-coherence regime could not be explored completely because of classical simulation constraints. Three of the sixteen Scenario 4 experiments did not complete within the maximum wall-clock allocation of seven days per run on the HPC environment and, therefore, did not produce results. The completed K=8 two-feature experiment (see Figure A16) already required approximately 89.1 h of execution time (see Table A8), while the remaining three configurations exceeded the available time budget. This limitation is a direct consequence of the classical simulation methodology: the Matrix Product State backend preserves increasingly large amounts of entanglement in the high-coherence regime, leading to rapidly growing computational costs. As a result, the largest-party, highest-coherence region of the parameter space remains only partially characterized in the present work. Consequently, the scalability considered in this work should be understood in two distinct senses. The protocol and satellite-enabled architecture scale formally through the resource laws discussed above, but the numerical demonstration is limited to the small and medium-size configurations that could be simulated with the available classical compute. In essence, the largest high-coherence configurations remain beyond the practical simulation budget used in this study.

Finally, several simplifying assumptions were adopted in the system-level model. The study focuses on a single-satellite architecture and does not explicitly model operational factors such as orbital scheduling, temporal satellite visibility windows, atmospheric turbulence and weather, pointing and tracking errors, acquisition time, background light, dynamic routing, queueing, finite link duty cycles, or hardware control overheads. In an operational satellite quantum network, these effects would reduce the temporal availability of usable entanglement and could increase the waiting time between distributed operations [17,19]. They would therefore mainly affect the achievable throughput of the protocol, rather than the structure of the distributed classifier itself. The communication layer should consequently be viewed as a tractable link-level abstraction for studying system-level constraints, rather than as a complete engineering model of a deployable satellite quantum network.

## 7. Conclusions & Future Work

### 7.1. Conclusions

We have presented a system-level feasibility study of satellite-enabled federated quantum machine learning based on a distributed distance-based quantum classifier. By combining a distributed quantum learning protocol, a hybrid space–ground entanglement-distribution architecture, and a noise-aware neutral-atom processor model, we were able to investigate how communication, computation, and data representation jointly influence application-level performance. The study therefore bridges three areas that are often considered separately in the literature: satellite quantum communication, distributed quantum computing, and quantum machine learning.

The results reveal that the performance of distributed quantum classification cannot be understood from hardware metrics alone. Instead, two qualitatively distinct failure modes emerge. In low-coherence regimes, decoherence dominates the distributed circuit and progressively destroys the quantum state required for interference-based classification. Depending on circuit complexity, this manifests either as near-random prediction behavior or as a strong collapse toward a single output class. As the coherence time increases, these hardware-induced effects are reduced, and a transition regime becomes visible, indicating that useful distributed quantum classification is governed by performance thresholds rather than a single sharp boundary.

A second and fundamentally different failure mode appears once the hardware becomes sufficiently coherent. In this regime, the quantum computation itself can preserve the overlap information required by the classifier, as demonstrated by the successful two-feature experiments. However, classification performance may remain poor when the selected feature representation is highly correlated or poorly conditioned. The observed failure of the four-feature configuration, even in the highest-coherence scenario, suggests that limitations originating from data geometry can dominate once hardware noise is sufficiently suppressed. This observation is consistent with known challenges of distance-based methods in redundant and highly correlated feature spaces, where distances and inner products lose discriminative contrast [37]. Rather than being solely a hardware problem, practical quantum machine learning must therefore also be viewed as a representation-learning problem.

An important outcome of this work is the identification of a regime in which the distributed quantum classifier remains operational despite the communication overhead imposed by a satellite-enabled architecture. For well-conditioned low-dimensional feature sets, increasing coherence leads to a clear recovery of classification performance, demonstrating that long-distance federated quantum learning is not fundamentally precluded by the distributed nature of the computation. At the same time, the observed scaling of entanglement resources and execution times highlights the critical role of quantum memories, entanglement generation rates, and communication latency in determining practical performance.

The study also exposes an interesting simulation paradox. The parameter regimes in which the distributed quantum system fails are comparatively easy to simulate classically because decoherence rapidly suppresses entanglement. Conversely, the regimes in which the quantum protocol begins to function correctly become increasingly expensive to simulate due to the preservation of quantum correlations. This observation strengthens the case for future execution on networked quantum hardware, where the most interesting operating regimes may ultimately become inaccessible to large-scale classical simulation.

Overall, the results support the view that satellite quantum communication can act as a meaningful enabling technology for future privacy-preserving distributed quantum applications. However, the path toward practical federated quantum learning requires progress across multiple layers of the technology stack. Improvements in processor coherence, entanglement-distribution infrastructure, quantum-memory performance, and communication efficiency must be complemented by learning-aware feature engineering and quantum data encoding strategies. The central conclusion of this work is therefore that the feasibility of distributed quantum machine learning is determined not by the communication network, the quantum hardware, or the learning algorithm in isolation, but by the interaction of all three. Successful large-scale deployments will require a systems-level co-design of networking, computation, and data representation.

### 7.2. Future Work

An important next step is the execution of the proposed protocol on actual networked quantum hardware. While the present study relies on classical simulation, experimental deployment on distributed quantum-network testbeds would enable direct evaluation of communication latency, memory decoherence, synchronization overheads, and non-local gate performance under realistic operating conditions [2,6]. Such experiments would be particularly valuable in the large-party, high-coherence regime, where classical simulation becomes increasingly challenging.

The results also highlight the importance of data representation in distributed quantum machine learning. Even when hardware limitations are overcome, classification performance may remain constrained by the geometry of the encoded feature space. Future studies should therefore investigate feature-selection and dimensionality-reduction techniques that are specifically designed for amplitude encoding and inner-product-based quantum inference. Approaches such as principal component analysis, correlation-aware feature selection, mutual-information-based ranking, and quantum metric learning may help construct feature spaces that are more compatible with quantum distance-based classifiers [13,37,38,39]. Moreover, future work in this area could investigate a more detailed parameter-sensitivity framework to systematically evaluate how feature selection, dataset composition, and different noise sources influence classifier accuracy.

A broader comparison between different quantum learning paradigms would also be valuable. The present work focuses on a distributed distance-based classifier, but alternative approaches, including quantum kernel methods, variational quantum classifiers, and distributed quantum neural networks, may exhibit different sensitivities to communication overhead, noise, and feature redundancy [38,40,41]. Such comparisons would help determine whether the observed data-limited failure mode is specific to distance-based classification or reflects a more general challenge in quantum machine learning.

The communication model itself can be extended substantially beyond the architecture studied here. Future investigations could incorporate multi-satellite constellations, repeater-assisted quantum networking, dynamic routing, satellite visibility windows, and orbital scheduling constraints. Integrating these effects would provide a tighter connection between application-level performance and the operational characteristics of global quantum communication infrastructures [14,15,16,17].

Additional opportunities exist at the interface between distributed quantum computing and quantum networking. The distributed classifier studied here relies heavily on non-local controlled operations and therefore consumes substantial entanglement resources. Techniques such as optimized non-local gate decompositions, entanglement recycling, communication-aware circuit synthesis, and circuit-cutting methods may reduce resource consumption and improve scalability [6,42].

Beyond distributed inference, a particularly interesting long-term direction is the study of federated quantum machine learning with distributed training. Extending the present framework from classification inference to collaborative model optimization would connect satellite-enabled distributed quantum computing to emerging research on quantum federated learning and privacy-preserving quantum artificial intelligence [43,44]. Understanding how noise, entanglement availability, and communication constraints jointly influence learning dynamics remains a largely unexplored research area.

## Figures and Tables

**Table 1:** Experimental configuration space explored in this study.

Parameter	Configuration
Number of parties (*K*)	{2,3,4,8}
Feature dimension (*M*)	{2,4}
Measurement budget ($N_{\mathrm{shots}}$)	{1000,10,000}
Scenario 1	$T_{1}=100$ µs, $T_{2}=50$ µs
Scenario 2	$T_{1}=20$ ms, $T_{2}=10$ ms
Scenario 3	$T_{1}=80$ ms, $T_{2}=40$ ms
Scenario 4	$T_{1}=10$ s, $T_{2}=1$ s

**Table 2:** Classification outcomes for the experiments shown in Figure 6, Figure 7 and Figure 8. *y* denotes the true label, $\hat{y}$ the predicted label, and $\Delta =|C_{0}-C_{1}|/N_{\mathrm{shots}}$ the decision margin.

Scenario	*K*	Features	Shots	*y*	$\hat{y}$	Correct	$C_{0}$	$C_{1}$	Δ
1	2	2	1000	0	0	Yes	509	491	0.018
1	2	4	1000	0	0	Yes	981	19	0.962
1	8	2	1000	1	0	No	505	495	0.010
1	8	4	1000	1	0	No	938	62	0.876
3	2	2	1000	0	0	Yes	502	498	0.004
3	2	4	1000	0	0	Yes	641	359	0.282
3	8	2	1000	1	1	Yes	496	504	0.008
3	8	4	1000	1	0	No	644	356	0.288
4	2	2	1000	0	0	Yes	772	228	0.544
4	2	4	1000	0	1	No	471	529	0.058
4	4	2	1000	0	0	Yes	982	18	0.964
4	4	4	1000	1	0	No	532	468	0.064

**Table 3:** Aggregate classifier performance across all shown experiments.

Scenario	Features	Correct/Total	Margin Range
1	2	5/8	[0.000, 0.062]
1	4	2/8	[0.850, 0.977]
2	2	2/8	[0.002, 0.026]
2	4	2/8	[0.860, 0.900]
3	2	4/8	[0.000, 0.018]
3	4	3/8	[0.272, 0.290]
4	2	7/7	[0.530, 0.964]
4	4	0/6	[0.004, 0.064]

**Table 4:** Circuit resources and simulation times for the experiments shown in Figure 6, Figure 7 and Figure 8. $d_{\mathrm{orig}}$ denotes the original circuit depth, $d_{\mathrm{transp}}$ the transpiled depth, and $\tau =d_{\mathrm{transp}}/d_{\mathrm{orig}}$ the transpilation blow-up factor.

Scenario	*K*	Features	Qubits	$d_{\mathrm{orig}}$	$d_{\mathrm{transp}}$	τ	Runtime
1	2	2	13	261	2410	9.2×	47 s
1	2	4	14	1029	3102	3.0×	1.2 min
1	8	2	28	2053	67,356	32.8×	6.5 min
1	8	4	29	8197	69,988	8.5×	9.4 min
3	2	2	13	261	2410	9.2×	33 s
3	2	4	14	1029	3102	3.0×	48 s
3	8	2	28	2053	67,356	32.8×	9.1 min
3	8	4	29	8197	69,988	8.5×	13.4 min
4	2	2	13	261	2361	9.0×	38 s
4	2	4	14	1029	2800	2.7×	49 s
4	4	2	19	1029	24,917	24.2×	50.6 min
4	4	4	20	4101	25,422	6.2×	1.3 h

**Table 5:** Projected resources for one 10000-shot classification query with four features and ten training points per party per class. The estimates are based on the same circuit construction used in the simulations.

Parties	$N_{\mathrm{pad}}$	Qubits	GHZ Pairs/Shot	GHZ Pairs/Query	Sequential Protocol Time	MPS Memory
8	128	29	2304	$2.3\times 10^{7}$	128 h	15 MB
16	256	46	4608	$4.6\times 10^{7}$	256 h	24 MB
32	512	79	9216	$9.2\times 10^{7}$	512 h	41 MB
64	1024	144	18,432	$1.8\times 10^{8}$	1024 h	76 MB
128	2048	273	36,864	$3.7\times 10^{8}$	2048 h	143 MB

**Figure A1:**
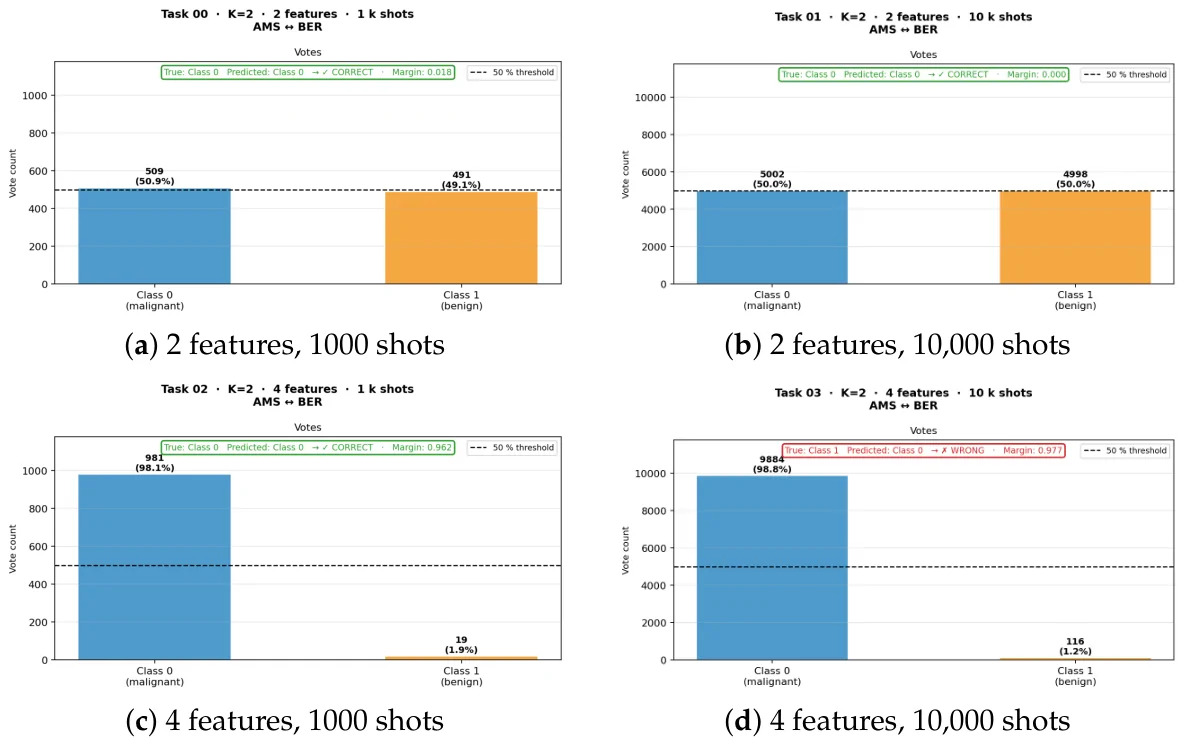
Results for K=2 parties (Amsterdam, Berlin).

**Figure A2:**
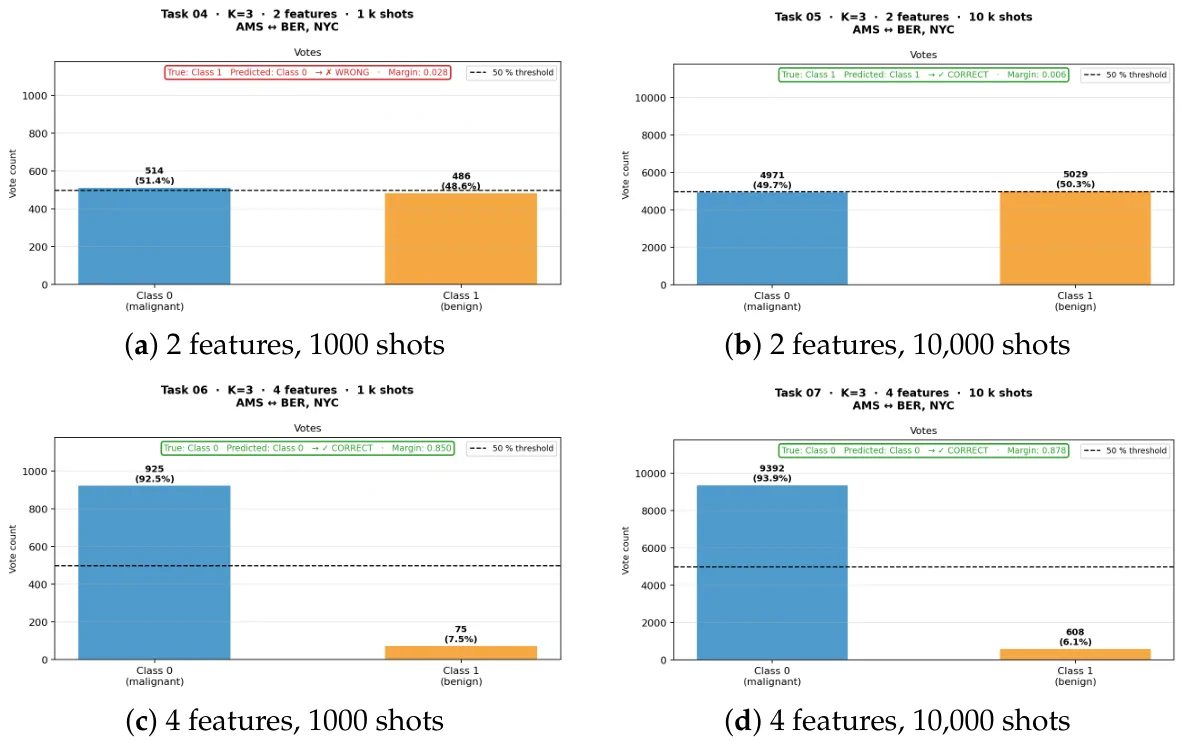
Results for K=3 parties (Amsterdam, Berlin, New York).

**Figure A3:**
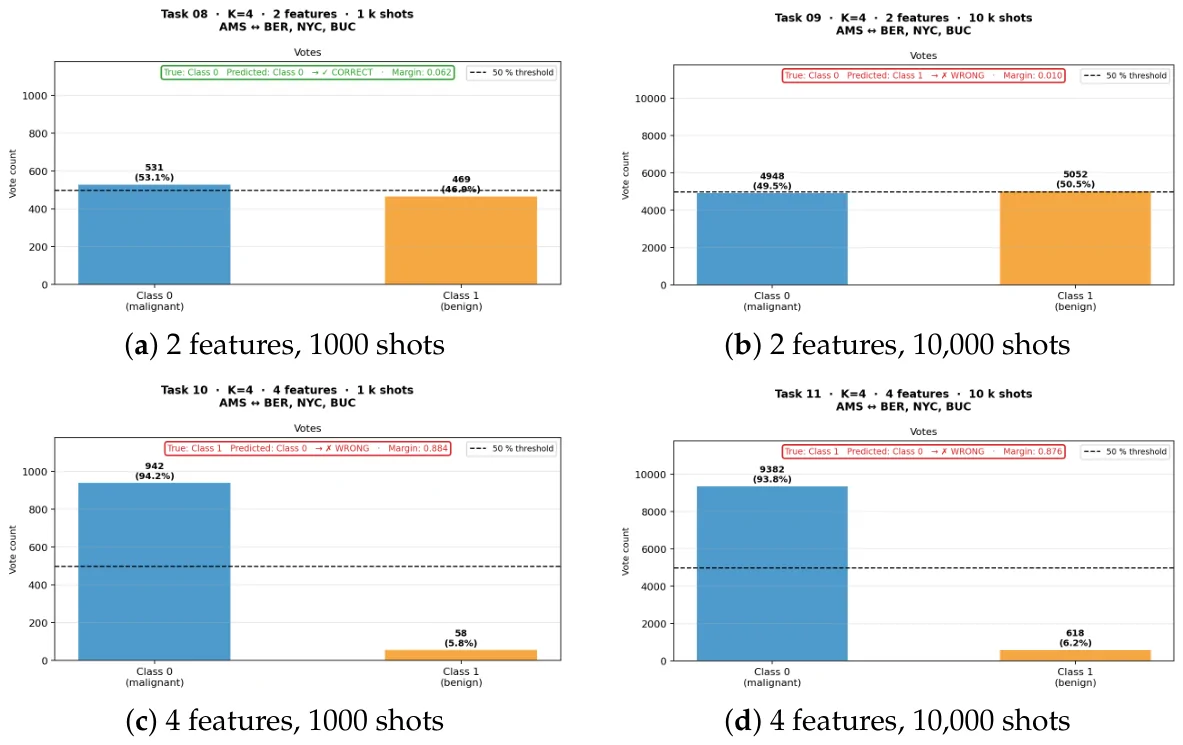
Results for K=4 parties (Amsterdam, Berlin, New York, Bucharest).

**Figure A4:**
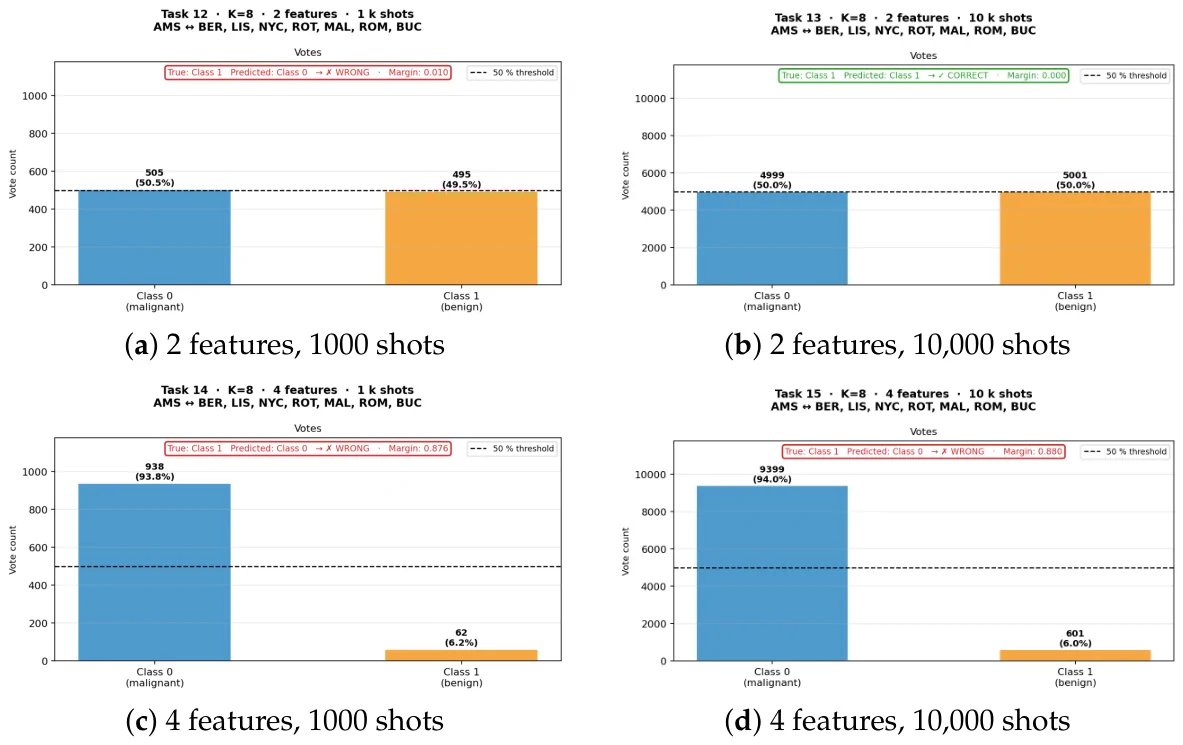
Results for K=8 parties (Amsterdam, Berlin, Lisbon, New York, Rotterdam, Málaga, Rome, Bucharest).

**Figure A5:**
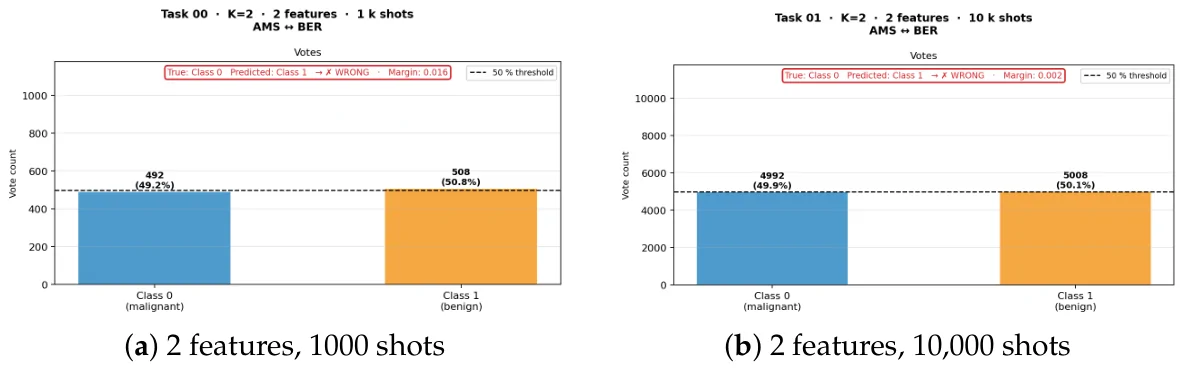

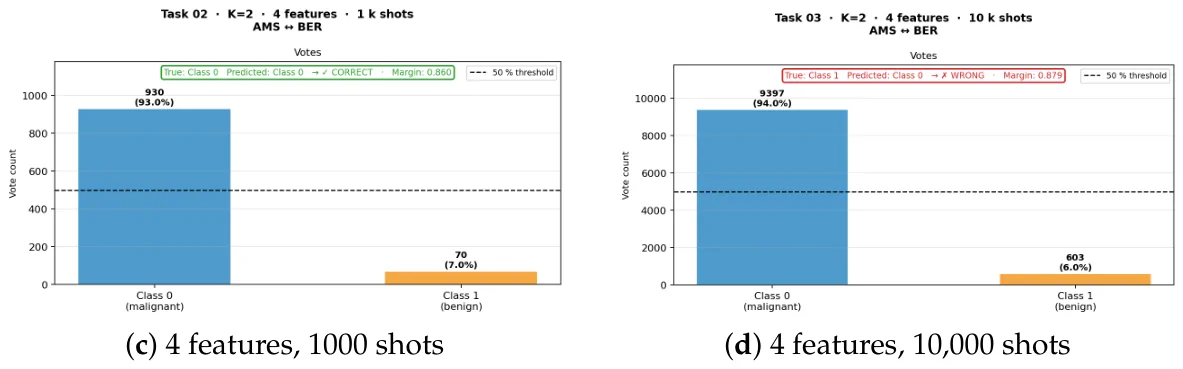
Results for K=2 parties (Amsterdam, Berlin).

**Figure A6:**
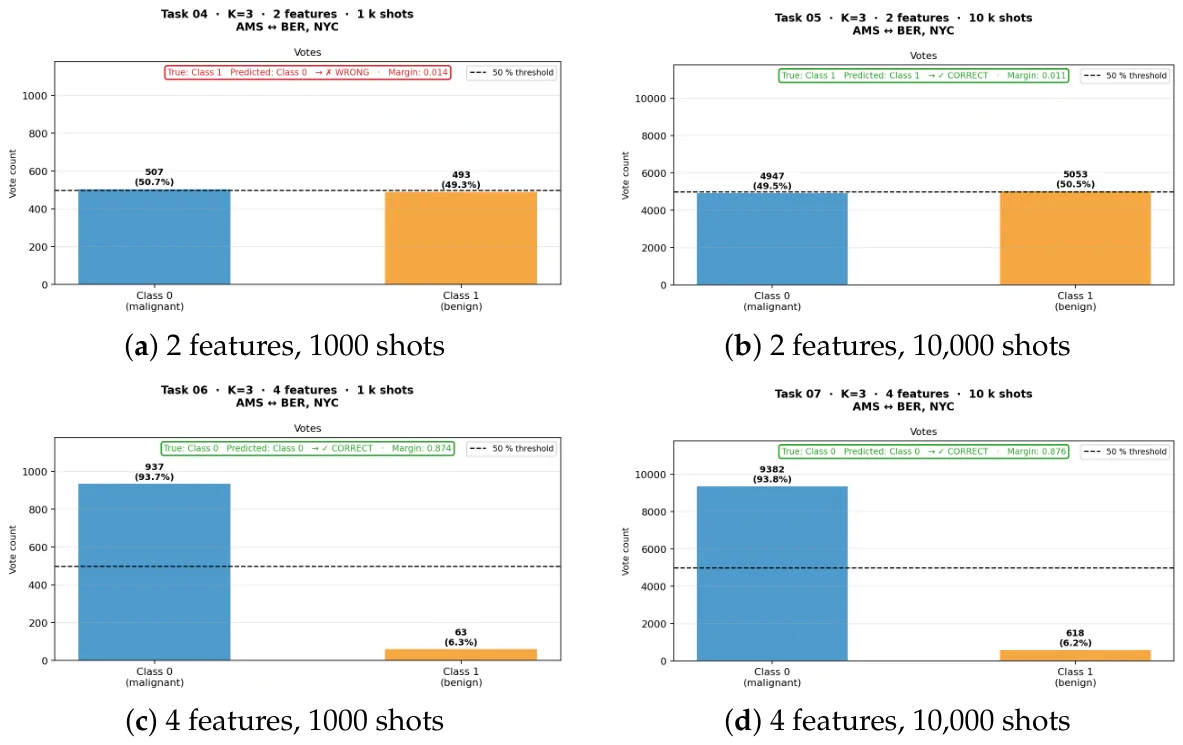
Results for K=3 parties (Amsterdam, Berlin, New York).

**Figure A7:**
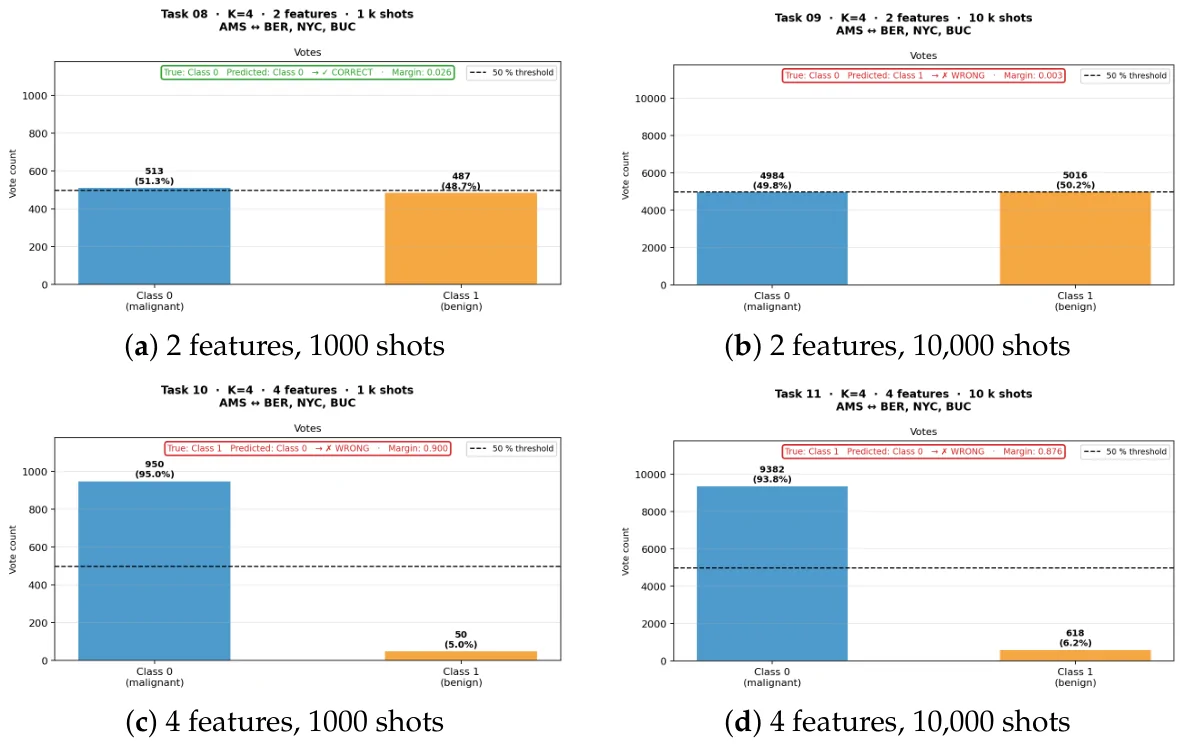
Results for K=4 parties (Amsterdam, Berlin, New York, Bucharest).

**Figure A8:**
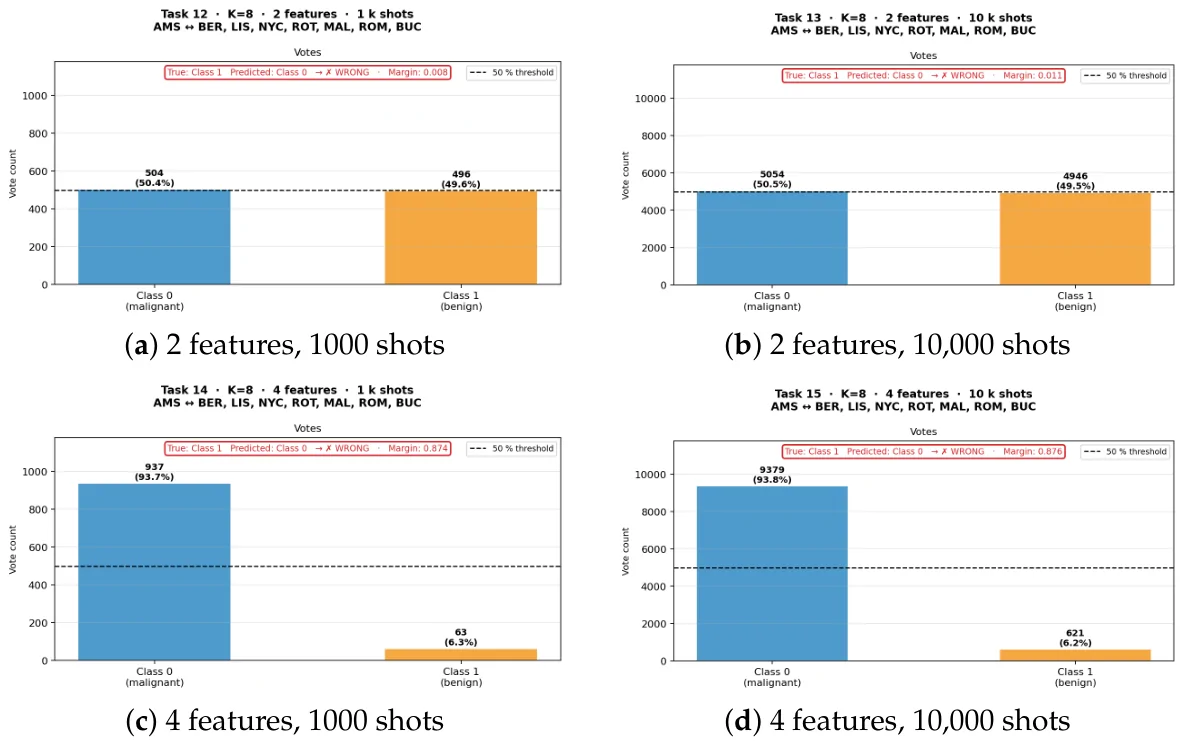
Results for K=8 parties (Amsterdam, Berlin, Lisbon, New York, Rotterdam, Málaga, Rome, Bucharest).

**Figure A9:**
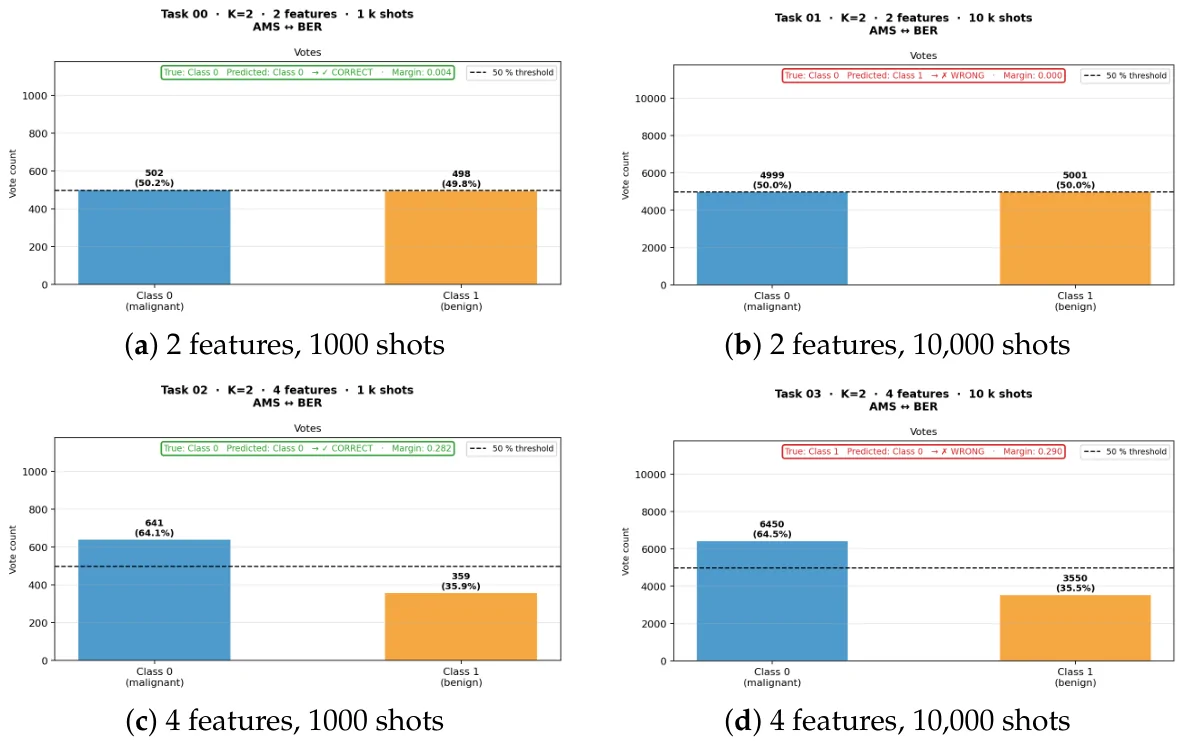
Results for K=2 parties (Amsterdam, Berlin).

**Figure A10:**
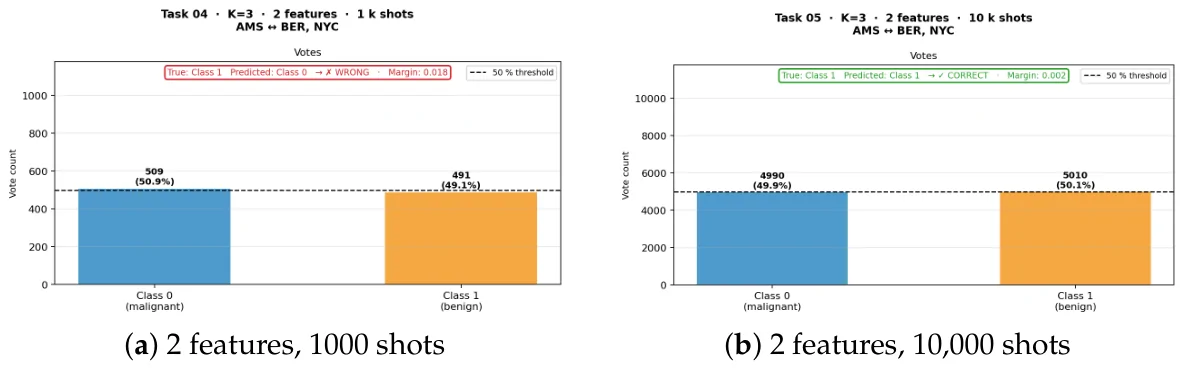

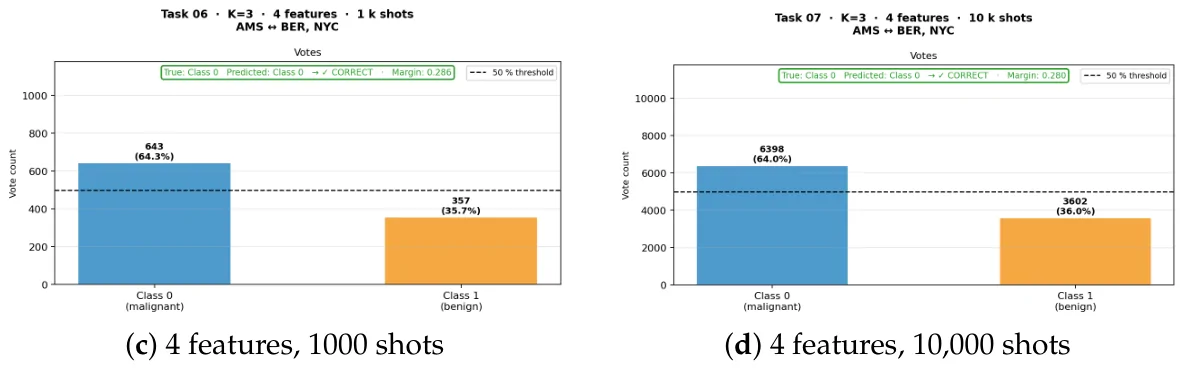
Results for K=3 parties (Amsterdam, Berlin, New York).

**Figure A11:**
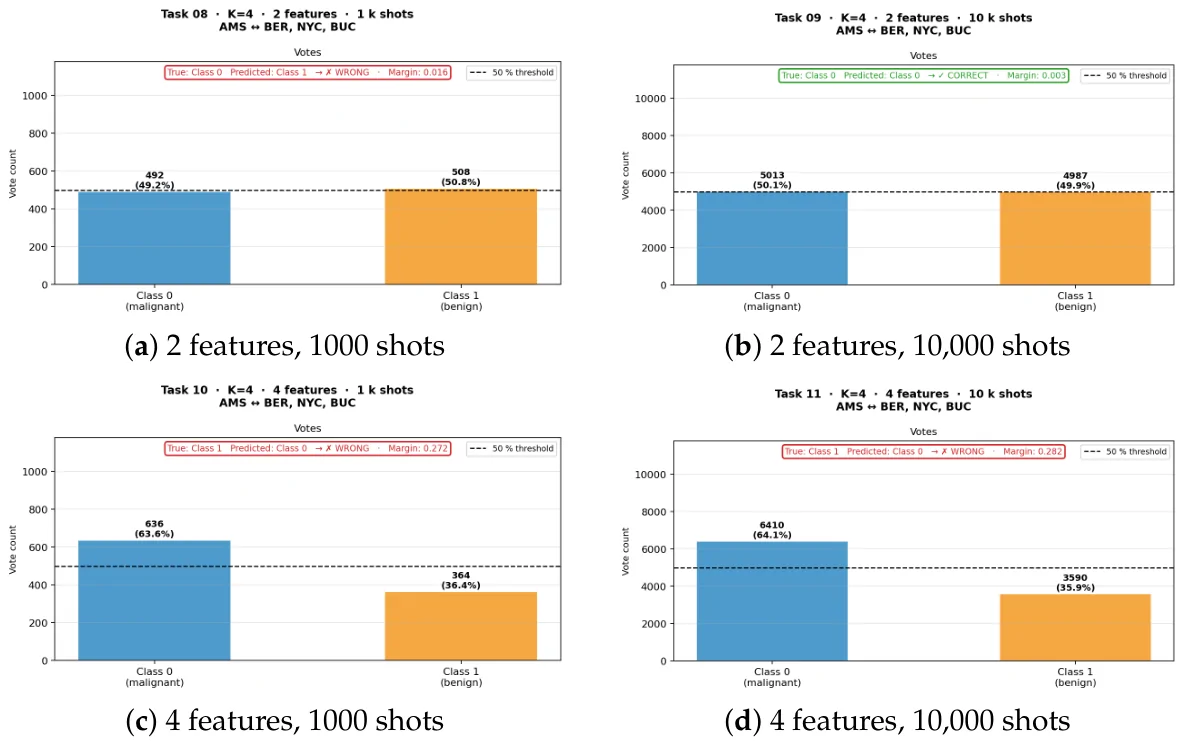
Results for K=4 parties (Amsterdam, Berlin, New York, Bucharest).

**Figure A12:**
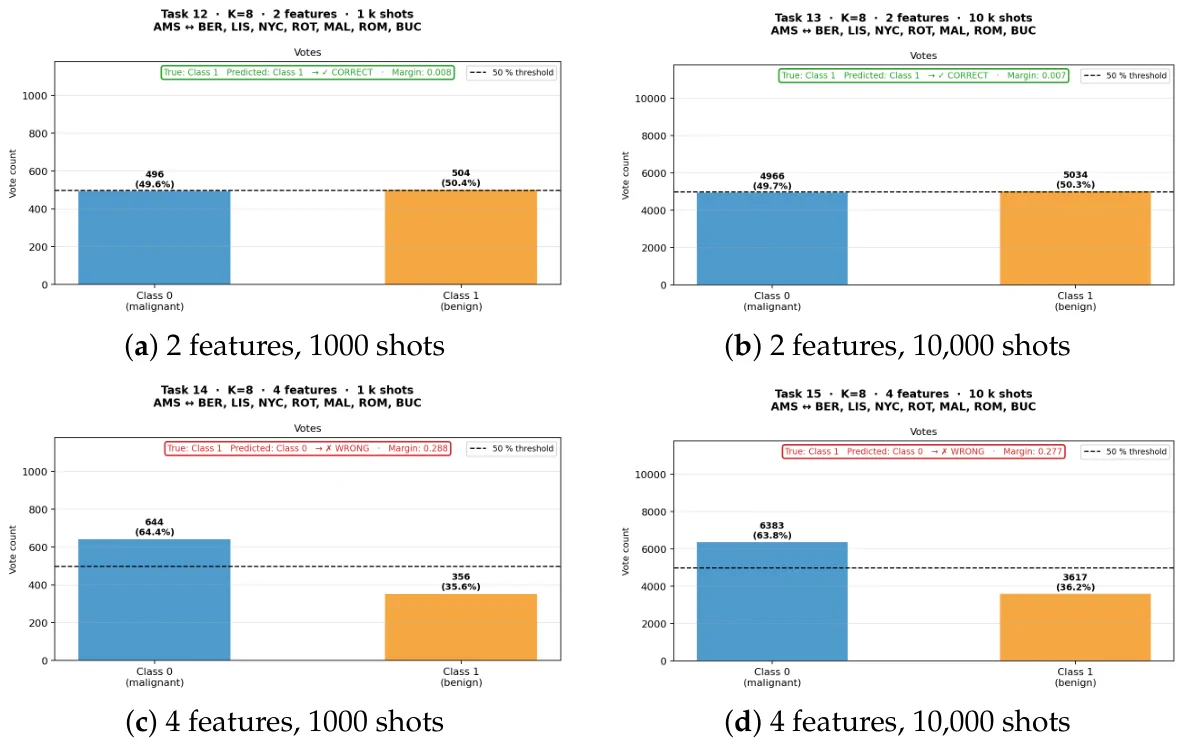
Results for K=8 parties (Amsterdam, Berlin, Lisbon, New York, Rotterdam, Málaga, Rome, Bucharest).

**Figure A13:**
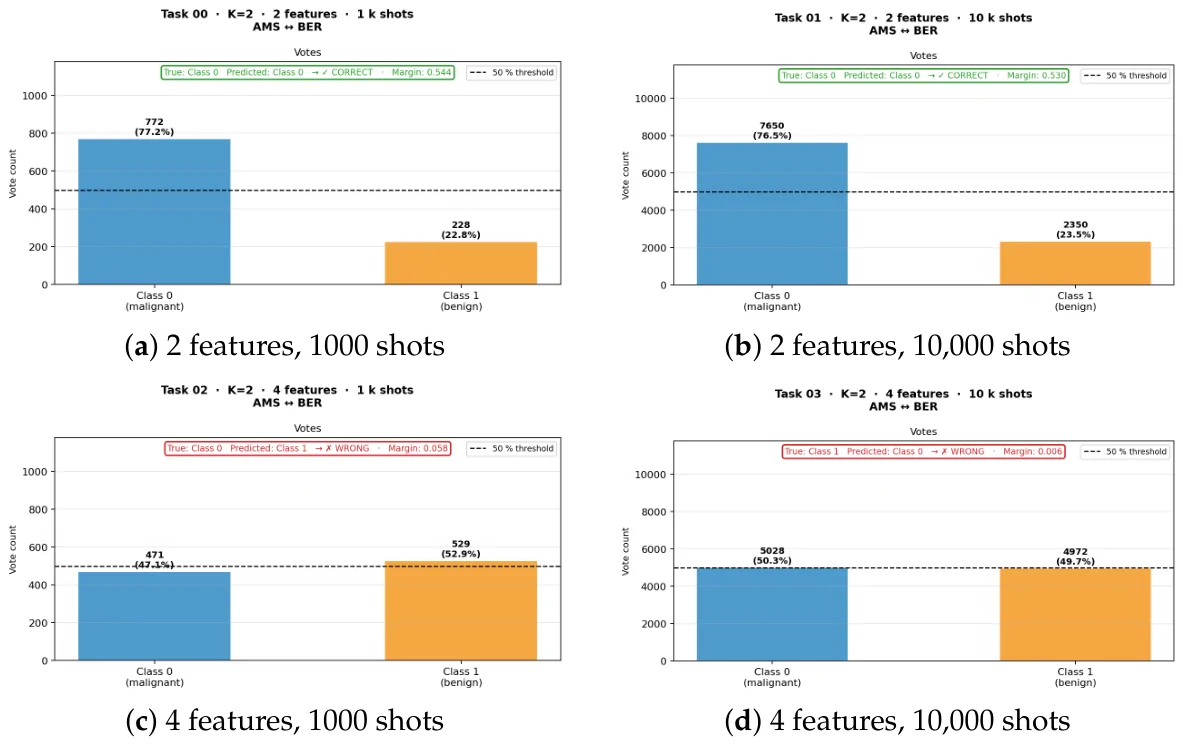
Results for K=2 parties (Amsterdam, Berlin).

**Figure A14:**
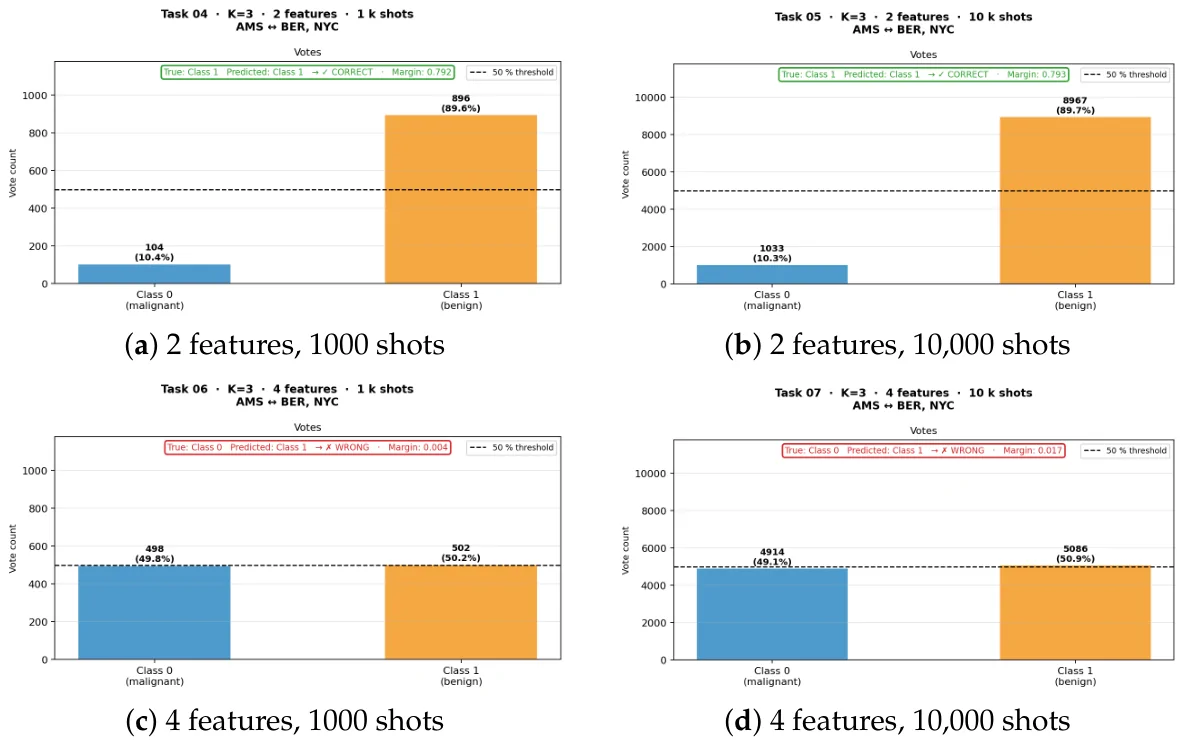
Results for K=3 parties (Amsterdam, Berlin, New York).

**Figure A15:**
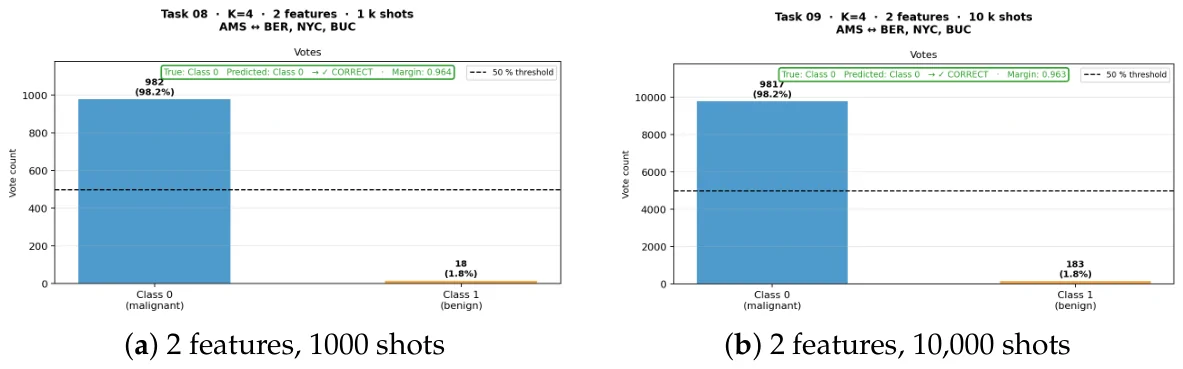

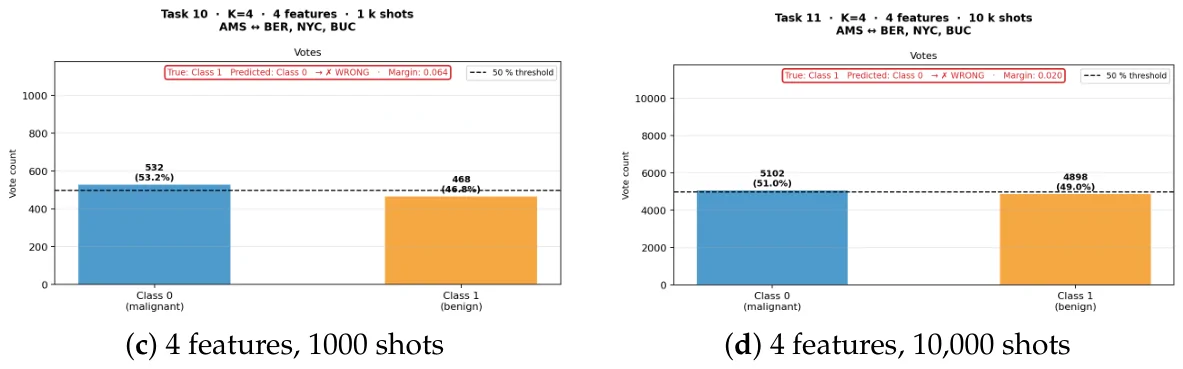
Results for K=4 parties (Amsterdam, Berlin, New York, Bucharest).

## Data Availability

The data used in this study are publicly available from the UCI Machine Learning Repository. Additional simulation outputs and scripts can be made available by the authors upon reasonable request.

## Abbreviations

The following abbreviations are used in this manuscript:

DQC	Distributed Quantum Computing
QPU	Quantum Processing Unit
DDBC	Distributed Distance-Based Classifier
QML	Quantum Machine Learning
GHZ	Greenberger-Horne-Zeilinger
BSM	Bell-State Measurement
MEO	Medium Earth Orbit
MPS	Matrix Product State
CPTP	Completely Positive Trace-Preserving
SPAM	State Preparation and Measurement

## Appendix A. Full Results

This appendix displays the results from all 4 coherence scenarios, across all number of parties, feature dimensions, and shot budget.

### Appendix A.1. Results Scenario 1: T1 = 100 µs; T2 = 50 µs

**Table A1:** Classification outcomes for the T1 = 100 µs; T2 = 50 µs scenario experiments. *y* = true class label (0 = malignant, 1 = benign), $\hat{y}$ = predicted label, $C_{0}$/$C_{1}$ = shot counts for class 0/1, $\Delta =\;|C_{0}-C_{1}|/\mathrm{shots}$ = decision margin.

# Parties	# Features	Shots	*y*	$\hat{y}$	Correct	$C_{0}$	$C_{1}$	Δ
2	2	1000	0	0	✓	509	491	0.018
2	2	10,000	0	0	✓	5002	4998	0.000
2	4	1000	0	0	✓	981	19	0.962
2	4	10,000	1	0	×	9884	116	0.977
3	2	1000	1	0	×	514	486	0.028
3	2	10,000	1	1	✓	4971	5029	0.006
3	4	1000	0	0	✓	925	75	0.850
3	4	10,000	0	0	✓	9392	608	0.878
4	2	1000	0	0	✓	531	469	0.062
4	2	10,000	0	1	×	4948	5052	0.010
4	4	1000	1	0	×	942	58	0.884
4	4	10,000	1	0	×	9382	618	0.876
8	2	1000	1	0	×	505	495	0.010
8	2	10,000	1	1	✓	4999	5001	0.000
8	4	1000	1	0	×	938	62	0.876
8	4	10,000	1	0	×	9399	601	0.880

**Table A2:** Wall-clock execution time per single classification query for the the T1 = 100 µs; T2 = 50 µs scenario experiments. In the *Correct* column, a correct prediction is depicted with a checkmark while an incorrect prediction is depicted with a cross.

# Parties	# Features	Shots	*t*
2	2	1000	47 s
2	2	10,000	3.2 min
2	4	1000	1.2 min
2	4	10,000	7.0 min
3	2	1000	1.6 min
3	2	10,000	9.0 min
3	4	1000	1.8 min
3	4	10,000	11.0 min
4	2	1000	2.6 min
4	2	10,000	16.8 min
4	4	1000	3.8 min
4	4	10,000	27.6 min
8	2	1000	6.5 min
8	2	10,000	45.7 min
8	4	1000	9.4 min
8	4	10,000	1.2 h

### Appendix A.2. Results Scenario 2: T1 = 20 ms; T2 = 10 ms

**Table A3:** Classification outcomes for the T1 = 20 ms; T2 = 10 ms scenario experiments. *y* = true class label (0 = malignant, 1 = benign), $\hat{y}$ = predicted label, $C_{0}$/$C_{1}$ = shot counts for class 0/1, $\Delta =\;|C_{0}-C_{1}|/\mathrm{shots}$ = decision margin.

# Parties	# Features	Shots	*y*	$\hat{y}$	Correct	$C_{0}$	$C_{1}$	Δ
2	2	1000	0	1	×	492	508	0.016
2	2	10,000	0	1	×	4992	5008	0.002
2	4	1000	0	0	✓	930	70	0.860
2	4	10,000	1	0	×	9397	603	0.879
3	2	1000	1	0	×	507	493	0.014
3	2	10,000	1	1	✓	4947	5053	0.011
3	4	1000	0	0	✓	937	63	0.874
3	4	10,000	0	0	✓	9382	618	0.876
4	2	1000	0	0	✓	513	487	0.026
4	2	10,000	0	1	×	4984	5016	0.003
4	4	1000	1	0	×	950	50	0.900
4	4	10,000	1	0	×	9382	618	0.876
8	2	1000	1	0	×	504	496	0.008
8	2	10,000	1	0	×	5054	4946	0.011
8	4	1000	1	0	×	937	63	0.874
8	4	10,000	1	0	×	9379	621	0.876

**Table A4:** Wall-clock execution time per single classification query for the T1 = 20 ms; T2 = 10 ms scenario experiments. In the *Correct* column, a correct prediction is depicted with a checkmark while an incorrect prediction is depicted with a cross.

# Parties	# Features	Shots	*t*
2	2	1000	33 s
2	2	10,000	1.8 min
2	4	1000	49 s
2	4	10,000	4.3 min
3	2	1000	1.1 min
3	2	10,000	5.3 min
3	4	1000	1.6 min
3	4	10,000	12.2 min
4	2	1000	2.6 min
4	2	10,000	18.9 min
4	4	1000	3.6 min
4	4	10,000	27.7 min
8	2	1000	6.7 min
8	2	10,000	45.8 min
8	4	1000	9.7 min
8	4	10,000	1.2 h

### Appendix A.3. Results Scenario 3: T1 = 80 ms; T2 = 40 ms

**Table A5:** Classification outcomes for the T1 = 80 ms; T2 = 40 ms scenario experiments. *y* = true class label (0 = malignant, 1 = benign), $\hat{y}$ = predicted label, $C_{0}$/$C_{1}$ = shot counts for class 0/1, $\Delta =\;|C_{0}-C_{1}|/\mathrm{shots}$ = decision margin.

# Parties	# Features	Shots	*y*	$\hat{y}$	Correct	$C_{0}$	$C_{1}$	Δ
2	2	1000	0	0	✓	502	498	0.004
2	2	10,000	0	1	×	4999	5001	0.000
2	4	1000	0	0	✓	641	359	0.282
2	4	10,000	1	0	×	6450	3550	0.290
3	2	1000	1	0	×	509	491	0.018
3	2	10,000	1	1	✓	4990	5010	0.002
3	4	1000	0	0	✓	643	357	0.286
3	4	10,000	0	0	✓	6398	3602	0.280
4	2	1000	0	1	×	492	508	0.016
4	2	10,000	0	0	✓	5013	4987	0.003
4	4	1000	1	0	×	636	364	0.272
4	4	10,000	1	0	×	6410	3590	0.282
8	2	1000	1	1	✓	496	504	0.008
8	2	10,000	1	1	✓	4966	5034	0.007
8	4	1000	1	0	×	644	356	0.288
8	4	10,000	1	0	×	6383	3617	0.277

**Table A6:** Wall-clock execution time per single classification query for the T1 = 80 ms; T2 = 40 ms scenario experiments. In the *Correct* column, a correct prediction is depicted with a checkmark while an incorrect prediction is depicted with a cross.

# Parties	# Features	Shots	*t*
2	2	1000	33 s
2	2	10,000	2.1 min
2	4	1000	48 s
2	4	10,000	5.0 min
3	2	1000	1.2 min
3	2	10,000	6.1 min
3	4	1000	1.9 min
3	4	10,000	11.5 min
4	2	1000	2.7 min
4	2	10,000	16.6 min
4	4	1000	3.8 min
4	4	10,000	36.8 min
8	2	1000	9.1 min
8	2	10,000	1.1 h
8	4	1000	13.4 min
8	4	10,000	2.0 h

### Appendix A.4. Results Scenario 4: T1 = 10 s; T2 = 1 s

**Table A7:** Classification outcomes for the T1 = 10 s; T2 = 1 s experiments. *y* = true class label (0 = malignant, 1 = benign), $\hat{y}$ = predicted label, $C_{0}$/$C_{1}$ = shot counts for class 0/1, $\Delta =\left|C_{0}-C_{1}\right|/\mathrm{shots}$ = decision margin.

# Parties	# Features	Shots	*y*	$\hat{y}$	Correct	$C_{0}$	$C_{1}$	Δ
2	2	1000	0	0	✓	772	228	0.544
2	2	10,000	0	0	✓	7650	2350	0.530
2	4	1000	0	1	×	471	529	0.058
2	4	10,000	1	0	×	5028	4972	0.006
3	2	1000	1	1	✓	104	896	0.792
3	2	10,000	1	1	✓	1033	8967	0.793
3	4	1000	0	1	×	498	502	0.004
3	4	10,000	0	1	×	4914	5086	0.017
4	2	1000	0	0	✓	982	18	0.964
4	2	10,000	0	0	✓	9817	183	0.963
4	4	1000	1	0	×	532	468	0.064
4	4	10,000	1	0	×	5102	4898	0.020
8	2	1000	1	1	✓	31	969	0.938

**Table A8:** Wall-clock execution time per single classification query for the T1 = 10 s; T2 = 1 s scenario experiments. In the *Correct* column, a correct prediction is depicted with a checkmark while an incorrect prediction is depicted with a cross.

# Parties	# Features	Shots	*t*
2	2	1000	38 s
2	2	10,000	2.0 min
2	4	1000	49 s
2	4	10,000	6.7 min
3	2	1000	2.8 min
3	2	10,000	20.5 min
3	4	1000	4.4 min
3	4	10,000	35.1 min
4	2	1000	50.6 min
4	2	10,000	7.3 h
4	4	1000	1.3 h
4	4	10,000	11.5 h
8	2	1000	89.1 h
